# Older Than Genes: The Acetyl CoA Pathway and Origins

**DOI:** 10.3389/fmicb.2020.00817

**Published:** 2020-06-04

**Authors:** William F. Martin

**Affiliations:** Institute for Molecular Evolution, University of Düsseldorf, Düsseldorf, Germany

**Keywords:** origin of life, bioenergetics, hydrothermal vents, autotrophic origins, evolution of pathways

## Abstract

For decades, microbiologists have viewed the acetyl CoA pathway and organisms that use it for H_2_-dependent carbon and energy metabolism, acetogens and methanogens, as ancient. Classical evidence and newer evidence indicating the antiquity of the acetyl CoA pathway are summarized here. The acetyl CoA pathway requires approximately 10 enzymes, roughly as many organic cofactors, and more than 500 kDa of combined subunit molecular mass to catalyze the conversion of H_2_ and CO_2_ to formate, acetate, and pyruvate in acetogens and methanogens. However, a single hydrothermal vent alloy, awaruite (Ni_3_Fe), can convert H_2_ and CO_2_ to formate, acetate, and pyruvate under mild hydrothermal conditions on its own. The chemical reactions of H_2_ and CO_2_ to pyruvate thus have a natural tendency to occur without enzymes, given suitable inorganic catalysts. This suggests that the evolution of the enzymatic acetyl CoA pathway was preceded by—and patterned along—a route of naturally occurring exergonic reactions catalyzed by transition metal minerals that could activate H_2_ and CO_2_ by chemisorption. The principle of forward (autotrophic) pathway evolution from preexisting non-enzymatic reactions is generalized to the concept of patterned evolution of pathways. In acetogens, exergonic reduction of CO_2_ by H_2_ generates acyl phosphates by highly reactive carbonyl groups undergoing attack by inert inorganic phosphate. In that ancient reaction of biochemical energy conservation, the energy behind formation of the acyl phosphate bond resides in the carbonyl, not in phosphate. The antiquity of the acetyl CoA pathway is usually seen in light of CO_2_ fixation; its role in primordial energy coupling via acyl phosphates and substrate-level phosphorylation is emphasized here.

## Introduction

It is part of our human condition to want to know about the past, where things come from and ultimately how life began. Indeed, most human cultures have an origins narrative of some sort. Scientists are also a form of human culture, in the broad sense, and as such scientists also have origins narratives. However, just like the origins narratives of different cultures tend to differ, so do the origins narratives of different groups of scientists. Mathematicians tend to prefer stochastic or probabilistic models; physicists tend to prefer complicated models that gravitate toward problems of self-organization, whereas chemists tend to prefer models that focus on the synthesis and polymerization of RNA bases. Biologists, on the other hand, tend to find deficiencies with all such models, probably because biologists recognize that life is a very complicated action involving all of the above and more. Life is a set of chemical reactions that are set in motion by energy metabolism. Given a source of electrons, energy metabolism, carbon metabolism, and sufficient nutrients, life reacts to generate cells that produce more cells until one of the educts becomes limiting. Cells deposit protein as the main substance, RNA as peptide-condensing agents, and DNA as memory; they self-organize, and they generate populations as side products of energy metabolism, the main chemical reaction that runs all of the above. The self-organization property of cells is not obvious. Hansen et al.(2009, p. 1843) reviewed studies of entropy change measurements during growth; the entropy change in cells is always zero or close to zero because, as they succinctly explained, “cells are assembled in a spontaneous process.” That is, if a cell has what it needs to grow, it organizes environmentally available components into more of itself as an effortless byproduct of the exergonic growth process. Growth means energy conversion, placing energy metabolism and changes in Gibbs free energy (Thauer, 2015) at the center of the origins question, from the perspective of physiology.

## What Is Ancient?

**FIGURE 1:**
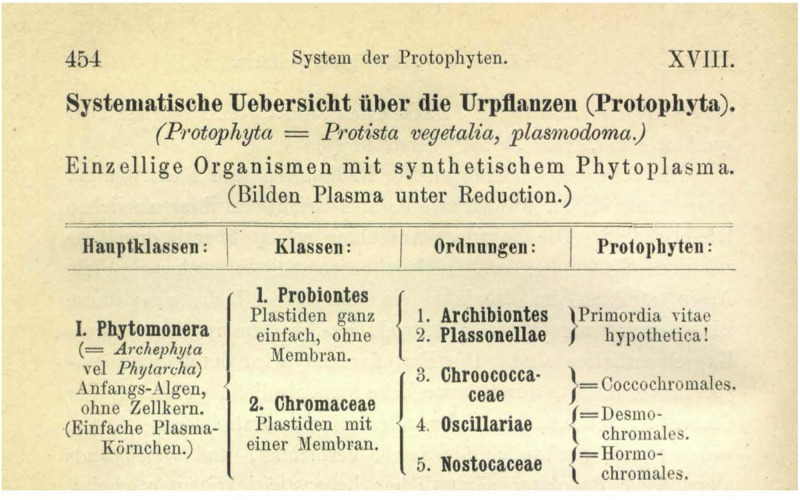
Haeckel’s proposal for Probiontes and Archibiontes. In my view, Probiontes would correspond to LUCA, whereas Archibiontes would correspond to the first free-living cells, which could have been cytochrome-lacking acetogens and methanogens that use the acetyl CoA pathway for H_2_-dependent carbon and energy metabolism (a grade not a clade). Note the term “Reduction” that Haeckel used to designate the growth of autotrophs (for heterotrophs, he used Oxidation). *Bilden Plasma unter Reduction:* Synthesize plasma reductively.

What do acetogens have to do with origins, and why include a chapter on the origin of life in a special issue about acetogens? The simplest answer is perhaps that biologists have always had an intuition that anaerobic bacteria capable of reducing CO_2_ are ancient. The idea that the first cells on earth were anaerobes and met their carbon needs from CO_2_ alone without the help of chlorophyll goes back 110 years. In 1902, Haeckel expressed the view that the first step in the origin of life (*Archigonie* he called it, from Greek *archae* ancient, *gone* seed) was the formation of an inorganic formative fluid (“*anorganische Bildungs-Flüssigkeit*”) containing the essential components, namely, carbonic acid, ammonia, and binary salts (“*Kohlensäure, Ammoniak, binäre Salze*”) (Haeckel, 1902, p. 361). As shown in Figure 1, Haeckel also saw the very first organisms as synthesizing their cell plasma reductively (“*Bilden Plasma unter Reduction*”), which today we would call autotrophy. Famous for his classifications, Haeckel placed these first organisms at the top of his system in the class Probiontes, represented by the first order Archibiontes, which contained only hypothetical types named *Primordia vitae hypothetica!* (Figure 1). Haeckel did not discuss the matter of origins much further in that book, although the exclamation point in *Primordia vitae hypothetica!*, possibly a punctuational singularity in the history of taxonomy, seems to underscore the importance of the issue.

**FIGURE 2:**
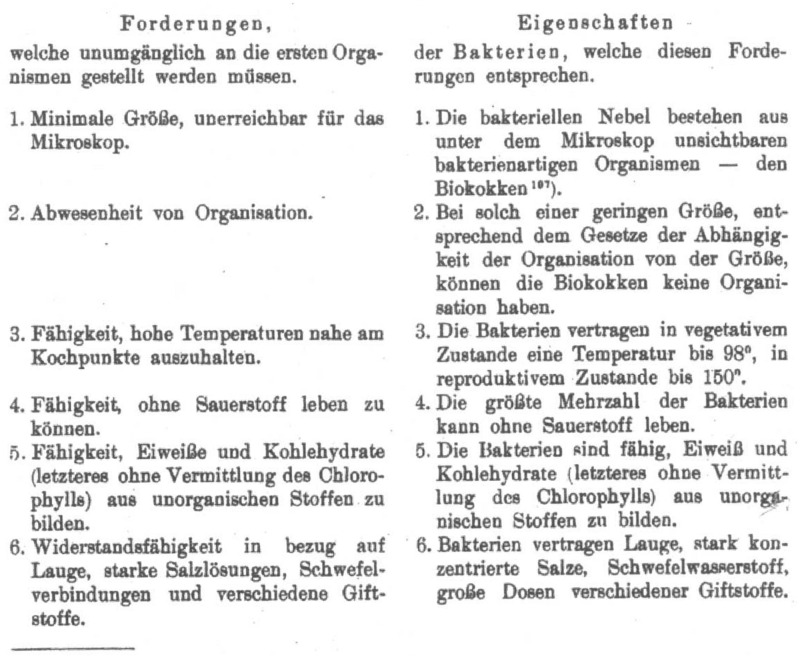
Mereschkowsky’s 1910 list of “Demands that inevitably must apply to the first organisms” (*Forderungen welche umumgänglich an die ersten Organismen gestellt wereden können*) and “Properties of bacteria that meet these demands” (*Eigenschaften der Bakterien, welche diese Forderungen entsprechen*). See text for translation of Demands 1–6. The demands are derived from his inference that life arose at a time when water on the young Earth’s surface was still hot, close to the boiling point (see text).

In 1910, Mereschkowsky took the issue of origins in the same direction but several explicit steps further. Mereschkowsky divided Earth’s early history into four phases, Epochs I–IV, as it pertained to origins. In the first epoch, the Earth had a fiery glowing surface; in the second, the fire had subsided, but the surface was still very hot, ≥100°C, and therefore dry; in the third Epoch, the surface was covered with boiling water (50–100°C); in the fourth, the water had cooled to less than 50°C (Mereschkowsky, 1910; p. 359). Based on observations of cells that grow at high temperatures, he then concluded that the first forms of life arose in Epoch III, as the Earth was covered in boiling water. Those first life forms furthermore had the following properties (translation by the author, the original German is in Figure 2): (1) a minimal size, inaccessible to the microscope; (2) a lack of organization; (3) the ability to survive temperatures close to the boiling point; (4) the ability to live without oxygen; (5) the ability to synthesize proteins and carbohydrates (the latter without the help of chlorophyll) from inorganic substances. [*Fähigkeit, Eiweiße und Kohlenhydrate (letzteres ohne Vermittlung des Chlorophylls) aus unorganischen Stoffen zu bilden.*]; and (6) resilience against alkaline solutions, concentrated salt solutions, sulfur compounds, and diverse toxins (Mereschkowsky, 1910; p. 359). Those six properties, taken together, are very close to what proponents of autotrophic origins at alkaline hydrothermal vents are saying today. This fifth criterion, autotrophy without chlorophyll, means chemolithoautotrophy in modern terms. Chemolithoautotrophic origins at H_2_-rich hydrothermal vents are concepts that tend to appeal to microbiologists because we can observe chemolithoautotrophs growing at hydrothermal vents today, and those modern environments are probably not much different than they were four billion years ago.

The idea that the first forms of life might have been phenotypically simple and chemically robust, arising in and inhabiting geochemically active environments, is intuitive conjecture. A similar conjecture shared by many biologists is that clues about the origin and early history of life are preserved in the biology of cells themselves and that some lineages of modern cells might be physiologically unchanged relative to the first life forms, which had to have been anaerobes, as Mereschkowsky (1910) and later Haldane (1929) were aware. Lipmann, who pioneered the concept of ATP- and energy-rich phosphate bonds as chemical currencies of energy in cells, was explicit about inferences from physiology when he wrote “Projecting backward makes it necessary to make assumptions which may seem difficult or perhaps impossible to verify. I think it might be possible to find links by looking more attentively for primitive evolutionary stages within the metabolic picture in the hope to apprehend there surviving metabolic fossils” (Lipmann, 1965, p. 273). The idea expressed there is not trivial. Biologists tend to hold that there are life forms still in existence today that have preserved aspects of physiology that were present in the first forms of life. This stands in contrast to much of the origins literature, where it is widely assumed and sometimes actively argued chemistry at origins is solely a process of generating RNA and has no connection at all to modern physiology (Orgel, 2008). Lipmann’s 1965 chapter is good reading; it makes a case for the greater antiquity of substrate-level phosphorylation over ion gradient coupled phosphorylation, the antiquity of ferredoxin, and, as a side note, the antiquity of RNA over DNA.

What were ancient life forms doing from the standpoint of energy metabolism? Electromagnetic radiation from the sun was long assumed to be energy source for energy at origins: Miller and Urey (1959, p. 247) stated that “At the present time the direct or indirect source of free energy for all living organisms is the sunlight utilized by photosynthetic organisms,” as expressed by Miller and Urey (1959, p. 247) as did Morowitz (1968, p. 79): “All biological processes depend on the absorption of solar photons and the transfer of heat to celestial sinks.” Of course, today we know that life in the crust and within hydrothermal vents proceeds in complete darkness, fueled exclusively by chemical energy provided by purely geochemical processes, without any need for sunlight whatsoever (Corliss et al., 1981; Baross and Hoffmann, 1985). That kind of harsh geochemical environment is much more in line with what Haeckel and Mereschkowsky had in mind. It is also the kind of environment where organisms that live from the reduction of CO_2_ with electrons from H_2_, acetogens, and methanogens have what they need for growth.

Acetogens and methanogens growing from geochemical H_2_ are not dependent on solar radiation. Following the lead of Decker et al. (1970) and advice from microbiologists knowledgeable of acetogen and methanogen physiology, I have made a case in recent years that acetogens and methanogens lacking cytochromes could have arisen from reactions of H_2_ and CO_2_ at hydrothermal vents ca. four billion years ago and have preserved to this day the founding physiology of the first free-living cells in the bacteria and archaeal lineages (Martin and Russell, 2007; Martin, 2008, 2012). Genomic reconstructions of the last universal common ancestor (LUCA) (Weiss et al., 2016) point very much in the same direction as to chemical experiments in the laboratory (Preiner et al., 2020). Microbiologists tend to understand that case; in textbooks, the theory is present (Madigan et al., 2019). In a hydrothermal origins scenario in which LUCA, confined to the site of the synthesis of its chemical constituents, had not yet progressed to the stage of a free-living cell, LUCA would correspond to Haeckel’s Probiontes (which in that case would possibly have priority over LUCA as a name). The first membrane-bounded cells to escape the vent, domain-founding acetogens and methanogens, would correspond to a grade comprising the first free-living cells, equivalent to Archibiontes. In that view, the differences that distinguish archaea from bacteria, including lipid and cell wall chemistry, would reflect their divergence from LUCA before the transition to the free-living state (Martin and Russell, 2003). That would correspond to a progenote organization of LUCA, something simpler than a free-living cell (Di Giulio, 2011; Weiss et al., 2016). Other views have it that LUCA was a fully fledged bacterium (Valas and Bourne, 2011), requiring complex evolutionary processes to account for the change in lipid and cell wall chemistry at the origin of archaea from bacterial roots (for a discussion see Sojo et al., 2014). There are a number of suggestions that LUCA was eukaryotic in organization, but from the standpoint of physiology, that possibility seems unlikely enough that we need not discuss it here. There might be suggestions out there that LUCA was an archaeon from which the bacteria would be derived, but this author is unaware of them. Haeckel’s designation of Archibiontes (Figure 1; from Greek *arkhaios* primitive) is an interesting ancient name for an ancient grade, the first free-living cells, which could have been acetogens and methanogens, based on their physiology (Decker et al., 1970).

## Acetogens Are Ancient

How far back can we trace the idea that acetogens might be living fossils from the origin of life? Lipmann (1965; p. 265) opined: “…the chlorophylls could scarcely be early in chemical evolution; if not for other reasons, this suggests that photosynthesis came relatively late, preceded by chemosynthesis already highly developed in anaerobic clostridia.” In a survey of energy conservation among anaerobes, and with keen attention to the evolutionary progression from cobalamin (ancestral) to chlorophyll via heme, Decker, Jungermann, and Thauer surmised that “From this point of view the methane-forming bacteria and the clostridia described in this article are closest to the primordial anaerobes” (Decker et al., 1970; p. 157) at a time before the recognition that methanogens are archae(bacteri)a. There might or might not be statements conjoining the evolutionary antiquity of acetogens and methanogens in earlier literature. Although Decker et al. (1970) were not talking about clostridia that grow from H_2_ and CO_2_, they were talking about methanogens that do, and they reported the thermodynamic values for both the acetogenic and the methanogenic reaction from H_2_ and CO_2_. The idea that acetogens and methanogens could harbor ancestral forms of prokaryotic energy metabolism has remained current in thoughts about ancient physiology because the more details that emerged from the investigation of enzymes, structures, and cofactors underpinning the pathway(s), the more ancient they appeared.

Chemists and biologists working on the acetyl CoA pathway agree that it is ancient, as a few quotes attest. Wood wrote “Perhaps we are uncovering some reactions used by primitive forms of life before the use of ATP was developed and before CO_2_ was used by the Calvin cycle” (Wood, 1991, p. 161). Ljungdahl surmised: “The autotrophic fixation of CO_2_ forming acetate is the most direct pathway for forming acetyl CoA, which may be the primary building block of life” (Ljungdahl, 2009, p. 20). For more than three decades, Fuchs has maintained that the acetyl CoA pathway is ancient: “The total synthesis of acetyl CoA fulfills most of the criteria postulated for an ancient pathway. Its distribution in only distantly related anaerobes (Archaebacteria and Eubacteria) […] and its unusual biochemistry are noteworthy. It requires the lowest amount of ATP. It is a versatile one-carbon and two-carbon assimilation path” (Fuchs and Stupperich, 1985, p. 245–246) or “The common ancestor of life was probably a chemolithoautotrophic thermophilic anaerobe… […] one attractive idea is that minerals catalyzed a primitive acetyl CoA pathway” (Berg et al., 2010, p. 11). Drake et al. concur: “The acetyl-CoA pathway and variants thereof appear to be important to primary production in certain habitats and may have been the first autotrophic process on earth and important to the evolution of life” (Drake et al., 2008). Ragsdale sees the situation similarly: “The isotopic fractionation pattern of anaerobic organisms using the Wood-Ljungdahl pathway suggests that they may have been the first autotrophs, using inorganic compounds like CO and H_2_ as an energy source and CO_2_ as an electron acceptor approximately one billion years before O_2_ appeared” (Ragsdale and Pierce, 2008. p. 1877). The editors of this volume do not dissent: “Acetogenic microorganisms may also have been among the first microorganisms” (Basen and Müller, 2017, p. 15) or, more recently, “The pioneer organism in a primordial world was probably a chemolithoautotrophic thermophilic anaerobe that employed the reductive acetyl CoA pathway” (Schoelmerich and Müller, 2019). Physiology tends to put acetogens at Square one of bacterial evolution.

Carbon isotope evidence consistent with the operation of the acetyl CoA pathway is found in rocks that are 3.8 billion years old (Ueno et al., 2006) and even 3.95 billion years old (Tashiro et al., 2017). The evidence for biological origin is founded in light carbon, an enrichment of ^12^C versus ^13^C. The alternative interpretation that those ancient carbon isotope signatures might reflect abiotic processes would suggest the existence of abiotic CO_2_ fixation prior to the origin of life, which would be compatible with theories for autotrophic origins. By about 3.5 billion years ago, stromatolites were present, suggesting the existence of photosynthetic communities, and many modern biochemical pathways had evolved (Nisbet and Sleep, 2001; Arndt and Nisbet, 2012). Nearly four billion years later, the acetyl CoA pathway is still the backbone of acetogen physiology (Wood, 1991; Ragsdale, 2008; Ljungdahl, 2009; Fuchs, 2011; Basen and Müller, 2017). The first organisms could have lived from H_2_ and CO_2_ in the geochemical setting of hydrothermal vents, fueled by the redox potential that exists between H_2_ from serpentinization and CO_2_ from the ancient oceans, the same redox potential that fuels growth of modern acetogens and methanogens (Preiner et al., 2018). The same reactions still fuel life for acetogens and methanogens in the deep crust today (Magnabosco et al., 2018). That kind of continuity from the first forms of metabolism into the physiology of modern cells is undoubtedly what Lipmann (1965) meant with the term “metabolic fossils.”

## Square Two

The first cells were likely autotrophs. What’s next? Comparative physiology of the six known CO_2_ fixation pathways among prokaryotes indicates that the acetyl CoA pathway is the most ancient, mainly because (i) it is linear rather than cyclic, (ii) it is the only exergonic CO_2_ fixing pathway, (iii) it is the only CO_2_ fixing pathway that occurs in archaea and bacteria (Berg et al., 2010; Fuchs, 2011; Hügler and Sievert, 2011), and (iv) it is a strictly anaerobic pathway, and it is replete with transition metal clusters (Ragsdale and Pierce, 2008). It is the only CO_2_ fixing pathway that operates via CO as an intermediate (Ragsdale, 2004), generating carboxyl groups from carbonyl, rather than reducing carboxyl groups, which is the key to its exergonic nature, as the other pathways expend energy to reduce carboxyl groups (Xavier et al., 2018). Furthermore, it is a pathway of both carbon and energy metabolism, which is an excellent starting point from which to undergo evolutionary specialized into distinct, dedicated pathways of independent carbon and energy metabolism (Martin and Russell, 2007). The linear nature of the pathway to acetate speaks for its antiquity over the other five cyclic pathways because they entail numerous stereochemically defined intermediates, whereas the condensation of a methyl group and CO generate no chiral centers in the CO_2_ fixation intermediates. The other five pathways are more restricted in distribution, the dicarboxylate/4-hydroxybutyrate cycle and the hydroxypropionate/4-hydroxybutyrate cycle occurring in archaea, the reductive citric acid cycle, the 3-hydroxypropionate bi-cycle, and the Calvin cycle occurring in bacteria (Berg et al., 2010; Fuchs, 2011).

Starting from the acetyl CoA pathway for carbon and energy, gluconeogenic carbohydrate pathways could have arisen (Say and Fuchs, 2010), accompanied by specialization of the pathway toward carbon assimilation supported by an energy metabolism that does not reduce CO_2_, as in sulfur reducers that oxidize H_2_, cell mass, or end products such as acetate or lactate (Liu et al., 2012; Schut et al., 2013; Sousa et al., 2013; Rabus et al., 2015). The invention of heme from corrin precursors (Decker et al., 1970) could have occurred in clostridial sulfate–reducing lineages (Martin and Sousa, 2015), where cytochromes are abundant. The closure of the horseshoe citric acid cycle into the reverse citric acid cycle in bacteria (Mall et al., 2018; Nunoura et al., 2018) likely marked the origin of the second CO_2_ fixation pathway. The first cells would have been dependent on H_2_ for carbon and/or energy metabolism, but in the absence of H_2_, only incremental physiological innovations were required for adaptation. The acetyl CoA pathway is reversible, as demonstrated in a sulfate reducer (Schauder et al., 1988), such that in the presence of low H_2_ partial pressures the pathway can support growth in the acetate oxidizing direction (Zinder, 1994; Hattori et al., 2005). Operation in the acetate oxidizing direction is likely an ancient property of the pathway, although the mechanism of coupling appears to be still unresolved.

The first specialized heterotrophs could have arisen using amino acid, nucleoside, and ribose fermentations of the cell mass left behind by H_2_-dependent autotrophs when their local geochemical supply of H_2_ subsided (Schönheit et al., 2016) because in the absence of H_2_ the fermentations become thermodynamically favorable. These innovations would have occurred in a world where primary production was dependent on geochemical H_2_ provided by serpentinization. Even the origin of photosynthesis is likely to have occurred at hydrothermal vents, taking root in mild thermal radiation rather than from harsh sunlight at the surface (Nisbet et al., 1995). Anoxygenic photosynthesis using a type I reaction center (linear electron flow) was likely key in that process, providing a means of primary production (ferredoxin reduction) that was no longer H_2_-dependent (Martin et al., 2018) and possibly involving zinc cytochromes as functional precursors of chlorophyll, the last of the tetrapyrroles to evolve (Decker et al., 1970). Chlorophyll-dependent light harnessing enabled the colonization of new niches and the establishment of new, ocean surface ecosystems by primary producers, paving the way to oxygenic photosynthesis (Allen, 2005; Fischer et al., 2016). In the archaea, metabolic innovations seem to often entail gene transfer from bacteria for physiological evolution (Nelson-Sathi et al., 2012, 2015; Martin and Sousa, 2016; Wagner et al., 2017).

## Three Problems: They Bifurcate, Pump, and Differ

The idea that acetogenesis and methanogenesis are ancient (Decker et al., 1970) is appealing. But is it robust, is it *belastbar* (German: able to bear weight)? One has to think things through in full, whereby the details can harbor demons. Three problems stand out.

One problem concerns the noteworthy aspect of acetogen and methanogen physiology that they require chemiosmotic coupling—ion gradient formation and ATP synthesis via a gradient-harnessing rotor–stator ATPase—for growth because there is not enough energy in the H_2_–CO_2_ couple to simultaneously support carbon assimilation and ATP synthesis via substrate-level phosphorylation. Their mechanisms of ion gradient formation entail flavin-based electron bifurcation (Herrmann et al., 2008; Buckel and Thauer, 2013). Electron bifurcation is, among other things, a very ancient mechanism to generate reduced ferredoxin from H_2_ (Müller et al., 2018). Ferredoxin is, in turn, the source of reducing power that acetogens and methanogens use for CO_2_ reduction because under standard physiological conditions the midpoint potential of the H_2_/H^+^ couple is not sufficiently negative to reduce CO_2_ (Herrmann et al., 2008; Buckel and Thauer, 2018; Müller et al., 2018; Peters et al., 2018). Electron bifurcation involves enzymes and cofactors. That would appear to complicate the idea that H_2_–CO_2_-dependent growth via acetogenesis and methanogenesis can be traced all the way back to exergonic reactions of CO_2_ in hydrothermal vents (Martin, 2012). Vents, however, offer a solution to this problem (see following section).

Moreover, the primitive forms of acetogens and methanogens that grow on H_2_ and CO_2_ for carbon and energy lack cytochromes and quinones (Thauer et al., 2008; Hess et al., 2014). For pumping, the acetogens that lack cytochromes and quinones use either an energy-converting hydrogenase (Ech) (Schoelmerich and Müller, 2019) or a ferredoxin-NAD^+^ oxidoreductase (Rnf) (Schuchmann and Müller, 2014). The methanogens that lack cytochromes and quinones pump via a methyl transferase that harnesses the energy in the transfer of a methyl group from a sulfur atom in a thiol to a nitrogen atom in a pterin to pump Na^+^ ions (Thauer et al., 2008). At first sight, this also appears to run counter to the idea that acetogens and methanogens (and their acetyl CoA pathway) are ancient. The fact that acetogens and methanogens growing on H_2_ and CO_2_ have to pump ions and use a rotor–stator ATPase in order to conserve energy would appear to squelch the idea. But that is not the case, because all prokaryotes (or their clades) use chemiosmotic coupling, and the rotor–stator ATP synthase is not only structurally conserved across bacteria and archaea (Grüber et al., 2014), it is as universal among prokaryotes and the genetic code itself (Sousa et al., 2013). The ATP synthase furthermore traces to LUCA in genomic reconstructions (Weiss et al., 2016). The problem that ensues is this: the principle and the enzyme of ion gradient harnessing, the ATP synthase, are conserved across acetogens and methanogens, but the mechanism of pumping is not. Vents also offer a solution to this problem (see following section).

Adding more complication to what seemed at the outset to be a fairly straightforward idea (carbon and energy metabolism via the acetyl CoA pathway in acetogens and methanogens is as ancient as rocks) is the circumstance that acetyl CoA pathway has two segments: one of which is conserved across acetogens and methanogens; the other is not. The two segments are CO-dependent acetyl CoA synthesis at carbon monoxide dehydrogenase/acetyl CoA synthase (CODH/ACS) (Ragsdale, 2008; Fuchs, 2011) and methyl synthesis from CO_2_ and H_2_. CODH/ACS is conserved; methyl synthesis is not (Sousa and Martin, 2014). Using electrons from H_2_ via ferredoxin, CODH/ACS reduces CO_2_ to CO at an FeNiS cluster and directs the CO through a tunnel in the enzyme to a second FeNiS cluster where it binds to a Ni atom as nickel carbonyl (Dobbek et al., 2001; Drennan et al., 2004; Doukov et al., 2008). The methyl synthesis branch is very different in acetogens and methanogens: the pathways use different cofactors (Maden, 2000), and the enzymes of the acetogen and methanogen pathways are not homologous (Sousa and Martin, 2014). To me, the methyl synthesis problem, or “the early formyl pterin problem,” has appeared to be the most severe (Martin and Russell, 2007; Martin, 2012), until recently. There is now a solution to this problem as well. All three solutions entail natural chemical properties of serpentinizing hydrothermal vents.

## Three Solutions, One Environment, and Catalyst

**FIGURE 3:**
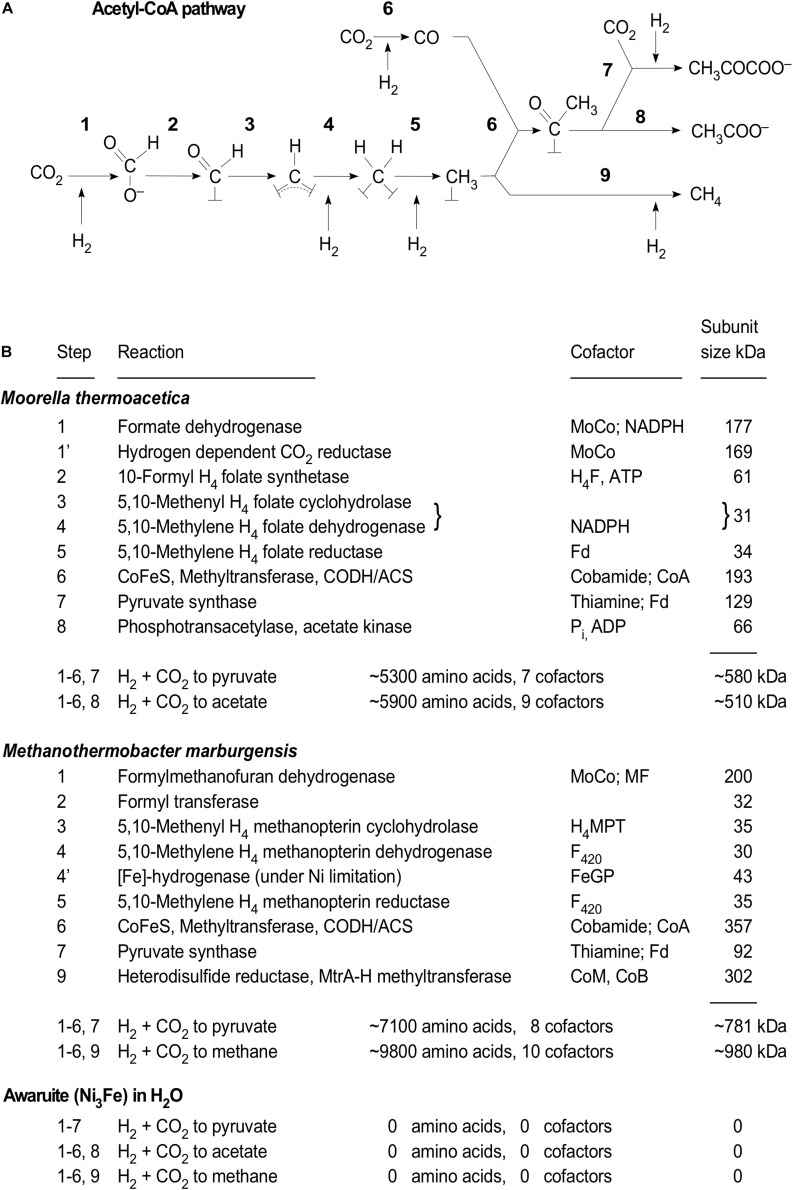
Chemical conversions in the acetyl CoA pathway. **(A)** The pathway as drawn by Fuchs (2011) but including the finding that in the methanogen pathway free formate is formed (Wagner et al., 2016). Modified from Preiner et al. (2020). The ligands of carbon represented as “⊥” are nitrogen atoms of pterin cofactors in the case of formyl, methenyl, methylene, and methyl groups in Reactions 3–5, cobalt and nickel atoms in the case of methyl group at Reaction 6, nickel atoms for the acetyl group at Reaction 6, or sulfur atoms in Reactions 8 (CoA) and 9 (CoM) (see Maden, 2000; Svetlitchnaia et al., 2006; Ragsdale and Pierce, 2008; Thauer et al., 2008; Ragsdale, 2009). **(B)** Cofactor requirements and the monomeric subunit size of the enzymes involved in the pathway in the acetogen *Morella* and the methanogen *Methanothermobacter* as summarized in the legend of Figure 1 of Fuchs (2011). Methyl syntheses in the bacterial pathway (also termed the Wood-Ljungdahl pathway) and the archaeal pathway differ in terms of their enzymology and cofactor requirements (Fuchs, 2011). The alternative [Fe]-hydrogenase (Huang et al., 2020), Reaction 4 of the methanogen, which is expressed under Ni limitation, is indicated. At the bottom of **(B)**, the end products of reactions catalyzed by awaruite (Preiner et al., 2020) are shown.

If acetogenesis and the acetyl CoA pathway are genuinely ancient (as in originating from rocks, water, and CO_2_), robust geochemical (prebiotic) solutions to the three problems outlined in the foregoing section—electron bifurcation, ion gradient formation, and methyl synthesis—are required. In Figure 3A, the acetyl CoA pathway is represented as a series of chemical conversions showing the oxidation state of carbon as it is reduced to a methyl group, to CO, to an enzyme-bound and cofactor-bound acetyl moiety and ultimately converted to acetate or methane in energy metabolism of acetogens and methanogens, or pyruvate in their carbon metabolism (Fuchs, 2011). The only difference in this depiction relative to Fuchs (2011) is the recent finding that free formate is generated in the methanogen pathway as revealed by the structure of the methanofuran dehydrogenase complex (Wagner et al., 2016), rendering the state of substrate carbon (although not its covalent ligands, generically represented as “⊥”) in the acetogen and methanogen pathways identical. Also, the pathway presented by Fuchs (2011) indicates the classical route of formate formation via NADPH-dependent formate dehydrogenase, whereby recently an alternative enzyme of formate synthesis in acetogens was reported, an H_2_-dependent CO_2_ reductase (Schuchmann and Müller, 2013) present in *Acetobacterium woodii* and *Thermoanaerobacter kivui*. It is conspicuous that, with the exception of CO and formate, substrate carbon is covalently bound either to cofactors or to active site atoms of the enzymes until released as acetate or methane (energy metabolism) or pyruvate (carbon metabolism).

Although not shown in Figure 3A, both pathways can assimilate environmentally available methyl groups via methyl transferases proximal to ACS or heterodisulfide reductase (Ragsdale, 2008; Thauer et al., 2008; Berg, 2011; Fuchs, 2011; Mayumi et al., 2016). Reductants in the pathway are indicated as H_2_, which is the environmental source of electrons during growth on H_2_ and CO_2_, although the reduced cosubstrates of the reactions are either NAD(P)H, reduced ferredoxin, or F_420_ (Fuchs, 2011). Figure 3B summarizes the names and molecular mass of the subunits of the enzymes of the acetyl CoA pathway from the acetogen *Morrella thermoacetica* and the methanogen *Methanothermobacter marburgensis*, compiled from the information in the legend of Figure 1 from Fuchs (2011). For the acetogen pathway, 10 enzymes with a subunit mass of more than 500 kDa and seven or nine cofactors are involved in the synthesis of pyruvate or acetate. Each of the cofactors (NADH, MoCo, thiamine, tetrahydrofolate, cobamide, CoA, ADP) has its own biosynthetic pathway of similar enzymatic demand. For the methanogen pathway, the situation is similar (>700 kDa), but perhaps more demanding because of the participation of additional cofactors including methanofuran, coenzyme B, coenzyme M, and F_420_, all of which have their own demanding biosynthetic routes (White, 2001; Graham and White, 2002).

Considering the intense enzymatic effort acetogens and methanogens invest into making pyruvate, acetate, and methane out of H_2_ and CO_2_ (Figure 3B), what initially looked simple starts looking like an insurmountable hurdle for prebiotic chemistry. We were therefore very surprised to find that formate, acetate, pyruvate, and methane are synthesized from H_2_ and CO_2_ under mild alkaline hydrothermal conditions (100°C, 24 bar, 16 h) using only one very simple and naturally occurring iron nickel compound, awaruite (Ni_3_Fe, both metals in the elemental zero valent state), as the catalyst (Preiner et al., 2020). That is, the entire function of the acetyl CoA pathway in carbon metabolism can be replaced by a piece of metal.

The findings of Preiner et al. (2020) fall very much in line with the old biochemical axiom that transition metal biocatalysis and transition metal sulfide clusters are ancient relicts from the early phases of chemical evolution (Eck and Dayhoff, 1966; Hall et al., 1971; Wächtershäuser, 1992; Volbeda and Fontecilla-Camps, 2006). The conversions of H_2_ and CO_2_ to formate, acetate, pyruvate, and methane shown in Figure 3A all involve transition metals. The hydrogenases of archaea and bacteria that channel electrons into CO_2_ reduction all have either Fe or Fe and Ni at their active sites (Thauer, 2011). Most, but not all, of those hydrogenase reactions reduce FeS clusters and then soluble redox cofactors or ferredoxins as the initial product of the hydrogenase reaction. The exception is the [Fe] hydrogenase (Hmd) of methanogens that lack cytochromes. Hmd transfers the electrons directly from H_2_, which is bound by the active site iron-guanylylpridinol (FeGP) cofactor, to an organic substrate, methenyl H_4_MPT, generating methylene H_4_MPT (Huang et al., 2020). This property of direct organic substrate reduction is so far unique among H_2_-activating enzymes (Huang et al., 2020). In Figure 3B, the biological reactions of the pathway involve catalysis requiring transition metals (Drennan et al., 2004; Dobbek, 2018), usually coordinated by sulfur (Sousa et al., 2018), sometimes coordinated by carbon (Martin, 2019), sometimes coordinated by nitrogen (Wongnate et al., 2016), or in the case of the Fe atom in Hmd, all three plus oxygen (the resting state VI of the mechanism; Huang et al., 2020). Catalysis and redox chemistry via transition metals and transition metal clusters, traditionally viewed as ancient, are the underlying theme of the acetyl CoA pathway.

That a single alloy, awaruite (Ni_3_Fe), can substitute for the entire enzymatic pathway (Figure 3) is either surprising or expected, depending on one’s standpoint. Awaruite is a typical constituent of serpentinizing systems. It is formed there by reduction of the divalent metals in host rocks by H_2_ from serpentinization (Krishnaro, 1964). A very similar spectrum of small organic products, but without methane detection, was obtained without H_2_, using native iron alone as both the catalyst and the reductant (Varma et al., 2018). Taken together, those findings indicate two things. First, the backbone of carbon and energy metabolism in acetogens and methanogens unfolds naturally from H_2_ and CO_2_ with a catalyst, Ni_3_Fe, which consists only of metal atoms and hence could not be simpler. Second, the findings provide concrete chemical evidence to support the view that the acetyl CoA pathway is not only ancient, as those who have worked on it always suspected, but it is older than the enzymes that catalyze it, either today or in the very first cells. The acetyl CoA pathway is older than the genes that encode its enzymes.

Given those observations, what are the solutions to the three problems? For bifurcation (generating low-potential reduced ferredoxin from H_2_), the solution is that serpentinization generates alkaline and H_2_-rich hydrothermal fluid. The midpoint potential of the low potential ferredoxins in acetogens and methanogens is on the order of −500 mV (Buckel and Thauer, 2013). The midpoint potential of H_2_ at pH 7 and 1 atm H_2_ is −414 mV. Flavin-based electron bifurcation provides a mechanism to generate low-potential ferredoxins for CO_2_ reduction (Buckel and Thauer, 2013; Müller et al., 2018). The midpoint potential of hydrothermal effluents stemming from serpentinizing systems can reach −900 mV (Suzuki et al., 2018). This introduces the possibility that organisms living in such environments might not need bifurcation for reduced ferredoxin synthesis (Sousa et al., 2018; Boyd et al., 2019). At origins, similar considerations apply. Using the Nernst equation for the dissociation of H_2_ into protons and electrons, the H_2_ partial pressures (1–10 atm), temperatures (100°C), and pH (8–10) used in the H_2_-dependent CO_2_-reducing reactions reported by Preiner et al. (2020) correspond to midpoint potentials in the range of −592 to −777 mV, sufficient for conversion of CO_2_ to organics or ferredoxin reduction if suitable catalysts are provided. The reducing power of serpentinizing systems in the Earth can, in principle, functionally substitute for electron bifurcation in cells by providing conditions sufficiently reducing to generate reduced ferredoxin (Sousa et al., 2018; Boyd et al., 2019), but whether this occurs in modern metabolism is so far unknown. In an origins context, however, it is now clearly demonstrated that methyl groups can be generated from H_2_ and CO_2_ under hydrothermal conditions using iron minerals without cofactors or enzymes (Preiner et al., 2020).

For the methyl synthesis problem, the solution is that in the presence of awaruite, or magnetite (Fe_3_O_4_) or greigite (Fe_3_S_4_), methyl synthesis from H_2_ and CO_2_ under hydrothermal conditions is facile (Preiner et al., 2020). Acetogens and methanogens have to invest energy in the form of ATP or reduced ferredoxin to generate methyl groups in the acetyl CoA pathway. That is the crux of the early formyl pterin problem. Modern serpentinizing systems emit methane in their effluent (Proskurowski et al., 2008; Etiope and Schoell, 2014), and methyl groups, as well as methanol itself, arise readily from H_2_ and CO_2_ in the presence of hydrothermal minerals as catalysts (Preiner et al., 2020). That indicates that the pathway could have gotten started with CODH/ACS as the first enzyme, operating with a geochemical supply of methyl groups, followed by independent origins (Sousa and Martin, 2014) of the unrelated methyl synthesis pathways of the bacteria and archaea. That would solve the energetic aspect of the early formyl pterin problem and explain why the chemistry of the pathway is so similar in bacteria and archaea (Figure 3A), but the enzymes and cofactors involved are so different. Enzymes do not shift equilibria; they just accelerate reactions that tend to occur anyway. The reactions were there first; the enzymes increased the reaction rates (Wolfenden, 2011).

For ion gradient formation, the solution is that the serpentinization process generates magnesium hydroxides from magnesium silicates (Bach et al., 2006; Russell et al., 2010; Sleep et al., 2011) with the result that the effluent of serpentinizing systems is generally alkaline, on the order of pH 9–11. This creates an ion gradient relative to the modern ocean, ca. pH 8 on the outside of the vent and ca. pH 9–11 on the inside. On the early Earth, the global ocean was more acidic, however, on the order of pH 6, because vast amounts of CO_2_ dissolved in it. Thus, in serpentinizing hydrothermal vents of the Hadean, alkalinity generated by serpentinization created a pH gradient, a proton (H^+^ ion) gradient, between the emerging effluent of the serpentinizing system and the ocean bottom water at the vent ocean interface of roughly three to four orders of magnitude. The polarity of the gradient is the same as that in modern cells: more alkaline on the inside than on the outside, generating a proton motive force from outside to in Martin and Russell (2007), Lane et al. (2010), Lane and Martin (2012)). That is about the same ΔpH range that biological systems generate in the process of ion pumping for the purpose of ATP synthesis. Such a geochemically generated ion gradient could have been harnessed by an ATPase at the origin of biochemistry, once genes and proteins had evolved (Martin and Russell, 2007; Martin, 2012). This solves the problem of how ion gradients arose before there were specific biochemical mechanisms to generate them: the first biochemical systems arose in environments where geochemical ion gradients were naturally existing (Russell and Hall, 1997; Martin and Russell, 2007; Lane et al., 2010; Sojo et al., 2014). Again, the polarity of ion gradients at alkaline hydrothermal vents (more alkaline on the inside than on the outside) is exactly the same as in cells (Martin and Russell, 2007; Lane and Martin, 2012). The evolutionary relationship of substrate-level phosphorylation (SLP) to chemiosmotic coupling is traditionally viewed as SLP coming first with chemiosmotic coupling coming later (Lipmann, 1965; Decker et al., 1970; de Duve, 1991; Ferry and House, 2006; Martin and Russell, 2007). Chemiosmosis enables energy conservation with substrates that provide less energy than necessary for SLP (Schuchmann and Müller, 2014).

## Phosphate and Energy

Although serpentinizing systems solve several problems in early physiological evolution, they present another: How, in terms of energetics, could genes and proteins (protein synthesis is ATP and GTP dependent) have evolved before a universal mechanism of ATP synthesis, ion gradient harnessing via a rotor–stator ATP synthase, which is a protein encoded by genes, had come to be? This question touches many facets of the origins problem, because it concerns the relationship between nucleic acids as molecular memory, peptides as catalysts, and the coupling of environmental energy to the polymerization reactions that generate both classes of biopolymers from their monomers. This harkens to the genetics-first versus metabolism-first discussion, which is widely thought to stem from the clash in the origins literature of the 1990s between Wächtershäuser’s ideas about pyrite-based metabolism contra efforts by proponents of an RNA world to quash them. As with most debates, the debate is older, as summarized yet again by Lipmann (1965; his opening statement on p. 259): “My motivation for entering into this discussion is an uneasy feeling about the tenet that a genetic information transfer system is essential at the very start of life. All efforts seem to be fixed exclusively on using presumably available energy sources, for example, electric discharges, for synthesizing nucleotides and amino acids and, therefrom, polynucleotides and polypeptides from various carbon–nitrogen sources. As I interpret it, the fascination with the two classes of compounds indicates the assumption that they are essential at the very outset. Being dissatisfied with this fixation on starting with the hen rather than with the egg, I have attempted to find alternatives. I am afraid that what I have to say will be just as much natural philosophy as necessarily most discussion on the origin of life need be at present. But try we must.” The concern from physiology that origins research is too focused on nucleic acids has tradition. In brief digression, note that Lipmann’s essay also discusses H_2_, H_2_S, and iron ions as sources of energy, in addition to a statement (p. 265) that will ring true to those interested in acetogens and methanogens: “I find it possibly of relevance that hydrogen activation, which would be involved here, is mediated by one of the more primitive catalysts, the recently discovered ferredoxin.”

Living cells are approximately 80–90% water by fresh weight. Nonetheless, a common criticism of hydrothermal systems as sites for biochemical origins is that they are full of water. This criticism typically comes from the genetics first camp and is based on the argument that, in aqueous solution, peptide bonds and the phosphoester bonds linking nucleotides will hydrolyze, leading to an inference that systems containing genetic material could not have evolved in a permanently aqueous environment (Bada and Lazcano, 2002; Orgel, 2008). Rather than prompt a conclusion that life must have evolved where there was no water (Benner and Kim, 2015), the thought about polymer hydrolysis should prompt the question: How does life deal with this problem? The answer is that life harnesses environmentally available energy and couples it to the synthesis of peptides and nucleic acids such that their polymerization is much faster than their hydrolysis. Let us assume for the sake of argument that it has always been this way. The hen to which Lipmann alluded was biopolymers; the egg was energy harnessing.

In biological systems, energy is mainly saved and spent in the currency of high-energy phosphate bonds: acyl phosphates, phosphoanhydrides, phosphoamides, carbamoyl phosphate, and phosphoenolate, all of which were known in 1941 (Lipmann, 1941). Phosphorus forms long covalent bonds with oxygen (Wald, 1962). This invites nucleophilic attack by water. The P–O and P–N bonds in the organophosphates of energy metabolism have high free energies of hydrolysis. In terms of Gibbs free energy under standard conditions at pH 7 (Δ*G*_0_′), hydrolysis of these high-energy phosphate bonds in metabolism releases on the order of −60 to −30 kJ/mol (Table 1). This release of free energy, if coupled to a slightly endergonic reaction, can make the reaction go forward. Coupled to many reactions, the hydrolysis of high-energy bonds makes the metabolism of a whole cell (life) go forward. That means that the high-energy bonds must constantly be resynthesized; otherwise, life comes to a halt. An *Escherichia coli* cell synthesizes roughly of 30 billion ATP (30 pg) or approximately 30 times its bodyweight (1 pg) per cell division (Akashi and Gojobori, 2002), a human synthesizes about a bodyweight of ATP per day.

**TABLE 1:** Free energy of hydrolysis for some biological compounds.

Phosphoenolpyruvate^a^	Δ*G*^o^′ = −62 kJ⋅mol^–1^
1,3-Bisphosphoglycerate^b^	Δ*G*^o^′ = −52 kJ⋅mol^–1^
Acetyl phosphate^a^	Δ*G*^o^′ = −43 kJ⋅mol^–1^
Creatine phosphate^a^	Δ*G*^o^′ = −43 kJ⋅mol^–1^
Carbamoyl phosphate^b^	Δ*G*^o^′ = −39 kJ ol^–1^
Acetyl CoA^c^	Δ*G*^o^′ = −32 kJ⋅mol^–1^
ATP (to ADP)^a^	Δ*G*^o^′ = −31 kJ⋅mol^–1^
Glucose-1-phosphate^a^	Δ*G*^o^′ = −21 kJ⋅mol^–1^
Inorganic pyrophosphate^d^	Δ*G*^o^′ = −20 kJ⋅mol^–1^
Glucose-6-phosphate^a^	Δ*G*^o^′ = −14 kJ⋅mol^–1^

*Values from Berg et al. (2015)^*a*^, Thauer et al. (1977)^*b*^, Buckel and Eggerer (1965)^*c*^, and Frey and Arabshahi (1995)^*d*^.*

As Lipmann (1965) pointed out, there are two mechanisms to make ATP. There is substrate-level phosphorylation (Lipmann called it fermentative phosphorylation or extract phosphorylation) in which a phosphate-containing carbon compound (Table 1) with a sufficiently high-energy bond phosphorylates ADP in a stoichiometric reaction. The other way to make ATP is the chemiosmotic mechanism of Mitchell (1961) with ion pumping plus ion gradient harnessing, which Lipmann called oxidation-chain phosphorylation because the mechanism of electron transfer to coupling via the ATP synthase had not yet been worked out. Lipmann concluded that substrate-level phosphorylation entailed a far simpler machinery; hence, it was the more ancient form of making high-energy phosphate bonds. From today’s perspective, that still seems correct (Martin and Thauer, 2017).

But Lipmann (1965) blazed a too seldom questioned trail in origins literature by suggesting that the participation of high-energy phosphate bonds in metabolism started with inorganic pyrophosphate (PP_*i*_) as the first chemical energy currency, coupled with his notion that SLP is more ancient than the ion gradient phosphorylation, which led to the idea that the entry of high-energy phosphate bonds into primitive metabolism came from high-energy phosphate bonds in phosphorus minerals in the environment. Although Lipmann’s idea of obtaining metabolic energy from pyrophosphate or polyphosphate minerals in the environment has a long tradition of acceptance in origins literature (Morowitz, 1992; Russell, 2006; Deamer and Weber, 2010; Pasek et al., 2017), the idea does not withstand inspection (see following paragraph). It furthermore distracts from the main issue at hand—the coupling of exergonic reactions of carbon reduction to early energy conservation (see following section).

One problem with PP_*i*_ or other environmental sources of preformed “high-energy” phosphorous bonds as the starting point for high-energy organophosphate bonds in metabolism is that it has no homolog in biology. That is not to say that there are no PP_*i*_-dependent reactions in metabolism—there are many. The point is that no biological systems are known to this author that access environmental PP_*i*_ or environmental polyphosphates as a source of energy. Stated another way, what cell will grow chemotrophically from PP_*i*_ or polyphosphate without the involvement of redox chemistry? None is probably the answer. The only examples from biology in which environmentally available phosphorous compounds play a role in energy metabolism involve phosphite as an electron donor in ion-pumping electron transport chains (Schink and Friedrich, 2000). The phosphite oxidizers are fascinating and important; they also clearly show that there is enough phosphite in the environment to support the existence of phosphite-reducing electron transport chains. However, that circumstance has nothing to do with Lipmann’s suggestion that environmental PP_*i*_ was a primordial energy source or an ancient energy currency. Another problem with PP_*i*_ that is equally pressing, if not more so, is that PP_*i*_ has a lower free energy of hydrolysis than glucose-1-phosphate (Table 1); it has low group transfer potential and is thus fighting a steeply uphill energetic battle in any effort to phosphorylate ADP for SLP or to activate any metabolic compound via formation of phosphoanhydride, phosphoester, or similar bonds. With the advantage of 50 years of hindsight following Lipmann’s 1965 suggestion, we now know that the most common function of PP_*i*_ in metabolism is not in energy metabolism, but immediate hydrolysis following reactions in which ATP is cleaved to AMP and PP_*i*_ so as to make the reaction irreversible under physiological conditions, such as in the activation of amino acids for translation (Berg et al., 2015).

## Acyl Phosphates Through CO_2_ Reduction

**FIGURE 4:**
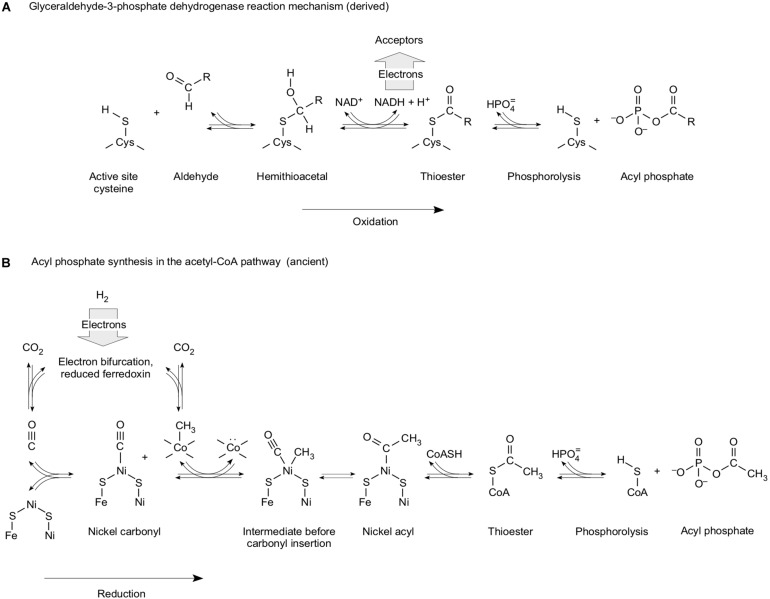
Energy conservation as high-energy phosphate bonds from carbon oxidation and carbon reduction [modified from Martin and Cerff (2017)]. **(A)** Mechanism of the D-glyceraldehyde-3-phosphate dehydrogenase reaction in the glycolytic (oxidative) direction to generate the mixed anhydride bond in 1,3-bisphospho-D-glycerate. *R* = CH(OH)CH_2_OPO_3_^2–^. The vertical arrow underscores the oxidative nature of the reaction in the energy-conserving direction. **(B)** Synthesis of acyl phosphate from H_2_ and CO_2_ as it occurs in the acetyl CoA pathway. Modified from Martin and Cerff (2017) and Martin and Thauer (2017). The reactions are drawn from data compiled in Svetlitchnaia et al. (2006), in Ragsdale (2009), in Fuchs (2011), and in Schuchmann and Müller (2014). Some methanogens can generate reduced ferredoxin via an energy-conserving hydrogenase, Ech, which does not entail bifurcation, but operates at the expense of an ion gradient, the generation of which demands bifurcation at the Mvh–Hdr complex (Thauer et al., 2008) in methanogens without cytochromes.

In SLP, ATP is usually synthesized during the oxidation of a reduced carbon compound (Lipmann, 1941; Decker et al., 1970; Martin and Thauer, 2017). When de Duve (1991) suggested that phosphorolysis of a thioester bond to form an acyl phosphate, as it occurs in the reaction mechanism of glyceraldehyde-3-phosphate dehydrogenase (Figure 4A), might mark the entry of phosphate into metabolism, he might have had the right kind of reaction mechanism, although it appears, from my perspective, that he put it in the context of the wrong upstream and downstream reactions. Leaning on the GAPDH reaction (Figure 4A), de Duve (1991) was suggesting that the oxidation of reduced carbon compounds present in the environment provided the source of energy. That is exactly what Wald (1964) had said 27 years prior about the origin of metabolism: life started from glucose fermentations (see Table 1 of Wald, 1964). Whatever happened to Mereschkowsky and autotrophic origins? Wald (1964) was suggesting that life started from glucose disproportionation (glycolysis and ethanol fermentation, where acetyl CoA is ultimately the electron acceptor), whereas de Duve (1991) was suggesting that sugars were oxidized with Fe^3+^ in the oceans being his preferred electron acceptor. The idea that there were enough free sugars lying around in the environment to provide an energy source for first life is still current in modern literature (Keller et al., 2014). More likely is the idea (Figure 2) that free sugars are made by cells from CO_2_ (Say and Fuchs, 2010; Fuchs, 2011). At any rate, following Lipmann’s lead, de Duve (1991) suggested that the acyl phosphate could be used to form PP_*i*_ as an ancestral metabolic energy currency, whereby PP_*i*_ will not work as an energy currency, as we saw above. The oxidation of preexisting reduced carbon compounds as a source of energy is couched in the outdated (Maden, 1995) concept of an organic soup, 100 year-old notion tracing to Oparin and Haldane concerning the origin of organic compounds in the first place. Soup was once popular (Garrison et al., 1951), but that was at a time before much was known about energy conservation in anaerobic autotrophs. That is, de Duve (1991) was deriving thioesters via analogy to heterotrophic metabolism.

In heterotrophic metabolism, SLP is always coupled to oxidation of reduced carbon substrates (Decker et al., 1970) except at the glycine reductase reaction of Stickland reactions, which generates acetyl phosphate (Andreesen, 2004). A carbon-oxidizing start to metabolism will not work, because a soup of substrates will be too complex to support energy metabolism (Schönheit et al., 2016), and because if strong oxidants are invoked, the accumulation of reduced organic compounds is thermodynamically unfavorable in the first place (Sousa et al., 2013). In autotrophy, carbon backbones unfold in a very natural and orderly manner that specifically generates the compounds of the acetyl CoA pathway (Figure 3).

Here, a point cannot be overemphasized. In SLP, the high-energy organophosphate bonds that are used to make ATP are formed by reactions of reactive carbon backbones with phosphate. It is not the reaction of reactive phosphorus compounds with unreactive organic substrates. It is the reaction of unreactive phosphate with reactive carbon compounds. The energy in the high-energy organophosphate bonds that are used for SLP (acyl phosphates, phosphoenolate) resides in carbon, not in phosphorus.

In autotrophic metabolism, acetyl phosphate can be synthesized for SLP during the process of CO_2_ reduction. SLP powered by CO_2_ reduction appears to be restricted to the acetyl CoA pathway. In that sense, Figure 3A (CO_2_ reduction) and Figure 4B (energy conservation) overlap well. In metabolism, phosphate is a cofactor, not a source of energy. It is an innocent bystander that forms a high-energy bond by its ability to perform nucleophilic attack of a reactive carbonyl. Does Figure 4B recapitulate a primordial reaction sequence coupling of CO_2_ reduction and energy metabolism? It well could be. Does that energy coupling work without enzymes? Almost.

The phosphorylation of ADP with acetyl phosphate is facile in the presence of Fe^3+^ (Kitani et al., 1991, 1995); acetyl phosphate can be readily generated from thioacetate and phosphate (Whicher et al., 2018). So far, no synthesis of acyl phosphates from CO_2_ and P_*i*_ has been reported. Would acyl phosphates from scratch be a big advance? It clearly depends on one’s point of view. It would help to explain how early energetic coupling was possible.

Findings from various disciplines tend to home in on the acetyl CoA pathway when it comes to origins. Investigations into ancient metabolism from the standpoint of modern metabolic networks are uncovering clues that converge on the acetyl CoA pathway (Goldford et al., 2017, 2019). Autocatalytic cycles can be identified within the metabolism of methanogens and acetogens (Xavier et al., 2020). Reactive chemical networks based on thioesters have been reported (Semenov et al., 2016). Starting from products of the acetyl CoA pathway, reactions of the reverse citric acid cycle take place in the absence of enzymes (Muchowska et al., 2017, 2019). Tryptophan is synthesized deep in geochemical systems (Ménez et al., 2018), which supports to the idea that reductive reactions at hydrothermal vents could have fostered life (Baross, 2018). Genomic reconstructions of LUCA indicate that it lived from gasses, using reactions and enzymes germane to the acetyl CoA pathway (Weiss et al., 2016). The enzymes of the acetyl CoA pathway are not only replete with transition metal sulfide centers (Russell and Martin, 2004), but they also contain half of all the carbon–metal bonds currently known in biology (Martin, 2019). Carbon–metal bonds are extremely rare in metabolism, and they are ancient. They occur only in enzymes that form the interface between metabolism and the gasses from which LUCA lived (H_2_, CO_2_, N_2_), or in enzymes and cofactors that transfer methyl groups, as shown in Figure 4B, or in cofactors that initiate radical reactions (Martin, 2019). They appear, to me at least, to be relicts of the catalysts that gave rise to primordial physiology.

## Amino Acyl Phosphates

And what good are acyl phosphates? They are energy currency, better than ATP. A look at Katchalsky and Paecht (1954, p. 6042) reveals that “In aqueous solution at room temperature, the phosphate anhydride of leucine polymerizes spontaneously to produce polypeptides of 3–20 amino acids.” Significant? Clearly, an energetic coupling of CO_2_ reduction to acyl or amino acyl phosphate synthesis would enable a great many biologically relevant reactions, such as peptide synthesis. One thinks of small molecule chemical networks of the kind that Kauffman had in mind (Xavier et al., 2020), and the words of Shapiro, who like Kauffman and many others was unconvinced that genetic material (the “hen” in Lipmann’s 1965 quote) came before the exergonic synthesis of the chemical components of which its monomers are comprised (the “egg”): “A more likely alternative for the origin of life is one in which a collection of small organic molecules multiply their numbers through catalyzed reaction cycles, driven by a flow of available free energy. Although a number of possible systems of this type have been discussed, no experimental demonstration has been made. The inclusion of a ‘driver’ reaction, directly coupled to the energy source, may lead to a solution” (Shapiro, 2006, p. 105). Spontaneous reactions that couple a biological driver reaction to synthesis of a biological energy currency cannot be far away. The overall reaction will probably look very much like acetogen energy metabolism, but with metals in place of enzymes. If carbon-based energy metabolism came first, carbon metabolism and, given a natural source of activated nitrogen (Preiner et al., 2018), the rest of metabolism would naturally follow.

## Patterned Evolution of Pathways, Not Retrograde Evolution

Prior to the publication of this article, a reader lamented that I seemed to be assuming retrograde evolution of pathways without saying so. This article is not about retrograde evolution of pathways; it is about the antiquity of a CO_2_ fixing pathway in the context autotrophic origins, which posit the outward evolution of pathways emanating from CO_2_, which is the opposite of retrograde evolution. Hence, there is clearly a gap in understanding between this author’s text and one reader’s subjective interpretation of same concerning the evolution of metabolism. Other readers might encounter the same problem, so it is worthwhile to briefly recapitulate retrograde pathway evolution and contrast it to the ideas in the present article.

The term “retrograde” comes from *retro* (Latin, backward) and *gradus* (Latin, step), or stepping backward. The concept of retrograde evolution of pathways traces to an article by Horowitz (1945), who argued that in the beginning there was a rich organic soup of the components from which cells are composed, amino acids bases and the like, in line with ideas of Oparin. These components, the products of modern pathways, became depleted through biological activity, creating pressure to synthesize them from their immediate biosynthetic precursors, which are presumed to exist in the soup as well. Notably, Horowitz assumes the existence of heterotrophic cells as the starting point of retrograde pathway evolution. Depletion of a given product Z creates pressure for the terminal enzyme in the pathway to be fixed so as to supply Z from precursor Y in a one-step pathway. In this way, the last enzyme in the pathway evolves first, catalyzing the reaction Y → Z. Subsequent depletion of Y generated, in turn, the pressure to supply Y from its preexisting precursor X, leading to evolution of the next to last enzyme in the pathway, catalyzing the reaction X → Y, yielding a pathway X → Y → Z, and so forth. In this way, pathways and metabolism as a whole evolved from the distal tips, the products, inward to the proximal core of central intermediates from which all products (amino acids and bases) are synthesized.

From tips to root means backward steps in evolution along the pathway relative to the biosynthetic direction, hence retrograde, although Horowitz did not use that word. Horowitz required the pathway evolving species to be heterotrophic for the compound in question, or in modern terms auxotrophic for all pathway products, taken across all pathways. Note that Horowitz’s model starts with organisms, species that already are alive, such that the retrograde model describes a process of inward biochemical pathway growth in a world where genes and organisms already exist in an organic soup having all intermediates and end products of a modern metabolic map in ample supply. A related concept is that of Ycas (1974), who suggested that gene duplications for an initially small number of enzymes of relaxed substrate specificity gave rise to toward a larger collection of enzymes each having higher substrate specificity. The theories of Horowitz and Ycas concern the vector of gene and enzyme evolution after the origin of organisms. The retrograde model of Horowitz explicitly posits that the first organisms were heterotrophs.

Autotrophic theories assume that the first organisms were autotrophs that obtained carbon from CO_2_. They differ from heterotrophic theories in that they assume that the organic molecules from which life arose were synthesized from CO_2_ and that the evolution of biochemical pathways to complex organics (amino acids and bases) thus recapitulates a vector of biochemical evolution that starts from CO_2_ and moves outward toward the tips, or products, of metabolism. In that regard, the main products that we see in metabolism today (amino acids and nucleic acids, together approximately 80% of the cell by weight) were not selected from a soup; rather, they were synthesized in a sequence of reactions such that they were the endpoints, not the starting points of biochemical evolution. In other words, heterotrophic origin theories operate via consumption of preformed products, whereas autotrophic origin theories operate via synthesis of products from CO_2_. In contrast to Horowitz (1945), autotrophic theories do not start with organisms. In contrast to Ycas (1974), they do not start with genes. Rather autotrophic theories entail the concept of chemical or physiological evolution before genes, starting from CO_2_. That is true for autotrophic theories of Mereschkowsky (1910), of Wächtershäuser (1992), of Shapiro (2006), and for autotrophic theories that are based on the acetyl CoA pathway (Martin and Russell, 2007).

Autotrophic theories have in common that they assume that life and metabolism started from CO_2_, hence that biochemical synthesis evolved from C_1_ compounds to C_2_ compounds to C_3_ and larger, such that the origin of metabolic networks was a process of growth from simpler to more complex (Wächtershäuser, 1992; Martin and Russell, 2007). Investigations of metabolic maps to uncover ancient cores and structures in metabolism are much in line with that view, as they uncover conservation surrounding an autotrophic core (Goldford et al., 2017; Xavier et al., 2020). The same core is uncovered in gene evolution studies that trace ancient genes to LUCA (Weiss et al., 2016, 2018). The most highly conserved core of that network, C_1_ → C_2_ → C_3_, or formate → acetate → pyruvate (Figure 3A), unfolds in simple laboratory reactors overnight from H_2_ and CO_2_ using hydrothermal minerals as catalysts (Preiner et al., 2020).

That said, what do autotrophic theories say about the evolution of genetically encoded biochemical pathways? Autotrophic theories assume that there was a process of chemical “evolution” before genes came into existence, whereby the term “evolutionary” in this context designates increases in complexity, not mutation or selection (processes connoting genes). Genes require the existence of the code; this article is not about the origin of the code. Once genes had arisen (we all have to agree that they did arise somewhere at some point), it is eminently reasonable to posit that the first genes to arise and evolve, in general, were those that anchored the genetic code in place, namely, aminoacyl tRNA synthetases (Carter and Wills, 2019). In terms of physiology, the first genes to arise and evolve were likely those that channeled a necessarily exergonic preexisting flux of carbon and nitrogen into components that reinforced the synthesis of genes and proteins (Martin and Russell, 2007). A survey of genes that trace to LUCA found precisely, namely, eight genes for aminoacyl tRNA synthetases and several enzymes involved in the acetyl CoA pathway, in nitrogen metabolism, in H_2_ assimilation, in cofactor biosynthesis, and in the synthesis of amino acids, bases, and modified bases (Weiss et al., 2016), which are essential for the code to operate (Becker et al., 2018; Weiss et al., 2018).

Of the autotrophic pathways known, only the acetyl CoA pathway occurs in both bacteria and archaea and enables ATP synthesis during CO_2_ fixation (Berg et al., 2010; Fuchs, 2011). The reverse oxidative citric acid cycle employing citrate synthase, the roTCA cycle, requires very little ATP input (Mall et al., 2018; Nunoura et al., 2018), but it does require the hydrolysis of one ATP per acetyl CoA generated, as opposed to supporting ATP synthesis while generating acetyl CoA. The interested reader is directed to Table S10 of Mall et al. (2018) for an excellent comparison of the overall energetics and ATP demand of CO_2_ fixing pathways in bacteria and archaea. In line with its favorable thermodynamics, the acetyl CoA pathway is also the only one of the autotrophic pathways known that has been shown so far to operate *in toto* without enzymes, as acetate and pyruvate are generated from H_2_ and CO_2_ by mineral catalysts alone (Preiner et al., 2020). Thus, from the standpoint of thermodynamics, it is the one from which to start (Figure 3). That would provide formate, acetate, and pyruvate, which in acetogens and methanogens spill over into the incomplete reverse citric acid cycle as the main source of carbon skeletons for biosynthesis (Martin and Russell, 2007; Fuchs, 2011; Goldford et al., 2017; Muchowska et al., 2019). The central proposition of autotrophic origins is that first biochemical pathways evolved outward from such a central core in a way that brought forth central intermediary metabolism from inorganically catalyzed non-enzymatic reactions. Inorganically catalyzed reactions came to be accelerated and channeled into metabolism-like conversions by accrual of organic catalysts (organic cofactors or their abiotic precursors) and then finally enzymes. In that sequence of events, the cofactors themselves could have been products of inorganic catalysis, with enzymes, however, being the products of genes.

This sequence of pathway evolution, namely, a sequence of CO_2_ assimilating reactions starting from inorganic catalysts, progressing to organic catalysts (cofactors), and on to enzymatic (gene encoded) catalysts, entails the very broad premise that the reactions of central metabolism leading to products (amino acids and bases) tend to take place naturally. Catalysts merely accelerate chemical reactions that tend to take place anyway, or the catalysts can alter the immediate products in the case kinetically controlled reactions. In that sense, the evolution of pathways under such a set of premises for autotrophic origins is prepatterned (Ger. *vorgezeichnet*; predrawn, sketched for the purpose of subsequent bolder drawing), or simply *patterned* by the natural reactions of carbon. Some readers will ask why not use the word palimpsestic instead of patterned. Palimpsestic, in addition to lacking all prosody, emphasizes the process of overbuilding or overwriting a prior state. Patterned, and more specifically *vorgezeichnet*, places the emphasis on the process of putting the original pattern, the ancestral state, in place. Patterned evolution of pathways emphasizes the process of generating the original pattern, namely, the natural reactions of organic compounds.

Thus, the concept of patterned evolution of pathways is the autotrophic counterpart of retrograde pathway evolution inherent to heterotrophic theories. Patterned pathway evolution has it that the reactions that comprise biochemical pathways were etched into the space of all possible chemical reactions according to kinetic and thermodynamic constraints, with environmentally available and novel synthesized catalysts bearing upon the relative rates of competing reactions. As pathways evolved forward, the spontaneous chemical reactions of preceding products determined the vector of evolutionary progression. The connections between products of different pathways, sometimes connecting pathway intermediates to generate new routes and products (widespread in cofactor biosynthesis) as one moves distal to the core, emerge as a natural result of patterned pathway evolution, as does the noteworthy thermodynamic stability of the main pathway end products, amino acids, and bases. Patterned evolution of pathways would readily explain why so many reactions in metabolism work well without enzymes (Martin and Russell, 2007; Keller et al., 2015; Muchowska et al., 2019; Preiner et al., 2020; Xavier et al., 2020).

## Life Is a Chemical Reaction

The same reader who was interested in retrograde evolution also suggested that I discuss an alternative theory that life evolved from large amounts of abiotically formed acetate. As it stands, there is no such theory out there in the literature to discuss, nor is there currently clear evidence for accumulation of abiotic acetate in large amounts, in contrast to clear evidence for abiotic accumulation of formate (Lang et al., 2018) and methane (Etiope and Schoell, 2014). Furthermore, if life started from acetate, the extraction of energy would be problematic. Acetate disproportionation to H_2_ and CO_2_ for energy metabolism generally requires a syntrophic partner that can scavenge the H_2_ so that the H_2_-producing reaction is exergonic (Hattori et al., 2005), meaning that for acetate disproportionation to work as the very first metabolism, the methanogen already has to be there, such that that acetate oxidation can hardly be the first metabolism, coming in second at best. Acetate disproportionation might, however, have arisen very early after acetogenesis (Martin and Russell, 2007). The alternative energy extraction route, acetate oxidation using high-potential terminal acceptors, is not an option at origins for the same reason that methane oxidation is not an option at origins: In the presence of high-potential acceptors, the reduced carbon compounds that need to accumulate for metabolism and life to arise in the first place are converted to CO_2_ (Sousa et al., 2013). The synthesis of acetate from H_2_ and CO_2_ is exergonic all by itself, as long as there is sufficient H_2_ and as long as there are no strong oxidants around. Acetate synthesis from H_2_ and CO_2_ is hence a good starting point for metabolic origins. Let’s take the idea one step further.

**FIGURE 5:**
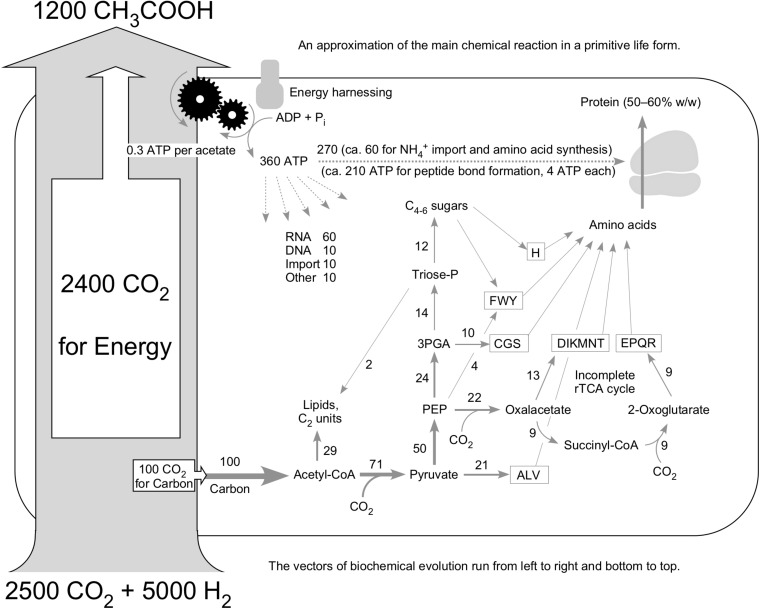
Idealized primordial metabolism for a hydrogenotrophic acetogen (see text). The carbon pathways are taken from Figure 6 of Fuchs (2011); the amino acid biosynthetic families are taken from Berg et al. (2015); the energy investment (dotted arrows at top) is taken from Stouthamer (1978); the 24:1 carbon ratio for energy metabolism versus cell mass accumulation is taken from Daniel et al. (1990); the ATP per acetate is taken from Müller et al., 2018). For fairness, Daniel et al. (1990) also reported that 0.3 mol of the carbon was unrecovered, which is neglected here. Numbers next to arrows in carbon pathways are from Fuchs (2011) and indicate the approximate percentage of flux. Relative width of carbon flux arrows is drawn roughly to scale, including the large gray arrow at left, to underscore the relative flux of material through the cell (large) versus the residue that remains (small). The acetyl CoA pathway roughly as indicated in Figure 4B resides within the large gray arrow and is not shown in detail.

Figure 5 summarizes metabolism in an ancient cell; an earlier and more preliminary version of the figure is found in Martin and Russell (2007). It conveys an approximation of the life process as a chemical reaction using the example of an acetogen. The starting point of Figure 5 is a study by Drake and colleagues (Daniel et al., 1990) in which they quantified the carbon flux through the cell as acetate and into cell mass for two acetogens. For *Clostridium thermoaceticum*, they found that during growth on H_2_ and CO_2_ approximately 0.1 mol of carbon accumulates as cell mass for each 2.4 mol of CO_2_ consumed. That is shown with the large gray arrow at the left of Figure 5. Thus, if we start with 2,500 atoms of carbon in CO_2_, approximately 2,400 of them are converted to acetate for energy metabolism, and approximately 100 of them go to cell mass. The fate of those 100 carbons in metabolism is given by Fuchs (2011), who provided a summary of carbon distribution in an idealized primordial metabolism based on the acetyl CoA pathway. The numbers next to the arrows in Figure 5 indicate the percent of acetyl moieties going toward C_2_ metabolism or being extended by further CO_2_ incorporation as given in Figure 6 of Fuchs (2011). Fuchs’ 2011 figure does not extend to amino acids but includes, probably by design, exactly the compounds from which the amino acid biosynthetic families (Berg et al., 2015) are derived—pyruvate, phosphoenolpyruvate, 3-phosphoglycerate, oxaloacetate, 2-oxoglutarate, and sugars. The amino acids are used to make protein, which comprises 50% to 60% of the cell’s mass.

The “additional” CO_2_ incorporations at pyruvate, oxaloacetate, and 2-oxoglutarate add up. Ten amino acids contain one additional carbon beyond the C_2_ starting unit, six amino acids have two additional carbons, and four amino acids have three additional carbons that are added from CO_2_. Had we started with 100 acetyl units (200 carbon atoms), pyruvate synthesis adds 71 more carbon atoms, oxaloacetate synthesis adds 22 more carbon atoms, and 2-oxoglutarate synthesis adds nine more (Fuchs, 2011), yielding 102 additional carbon atoms. Thus, per 100 carbon atoms from acetyl CoA, approximately 50 more are incorporated after acetyl CoA synthesis. The acetyl CoA pathway provides approximately two-thirds of the carbon, roughly one-third coming from subsequent incorporations.

To make protein, the amino acids have to be activated as aminoacyl tRNA, which hydrolyzes ATP to release PP_*i*_ at synthesis of the amino adenylate intermediate, requiring two ATP as input and then two steps of GTP-dependent ribosome movement, or four ATP per peptide bond (Berg et al., 2015). The energy for that comes from acetogenesis, which delivers approximately 0.27 or, rounded, 0.3 ATP per acetate, as Müller et al. (2018) worked out. For the 1,200 acetate produced, that yields approximately 360 ATP, which, if we consult Stouthamer (1978) regarding the rough distribution of energy costs across the cell, is enough to make approximately 52 peptide bonds. A smaller amount of ATP is required for RNA, DNA, and other things (Figure 5).

Keeping in mind that approximately only 60% of cell carbon goes to protein (cells are 50% carbon and 30% carbon in protein by weight), Figure 5 has it that 60% of the 150 carbon atoms assimilated per 1,200 acetate, or 90 carbon atoms can be directed toward peptide synthesis. But an average amino acid has five carbons, so that there is enough energy to make 52 peptide bonds but only enough carbon to make 18 amino acids. The available energy for peptide synthesis exceeds the available carbon for peptide synthesis by approximately a factor of three. Can that be right?

A three-fold excess of energy relative to protein cell mass seems odd at first sight, but in Figure 5, we have not considered maintenance energy or ATP spilling, which can be substantial. Stouthamer (1973) showed that the theoretical maximum yield for cell mass for *E. coli* (on the order of 28 g per mol ATP synthesized) is approximately three times the measured value (approximately 10 g per mol ATP synthesized). *Escherichia coli* cells synthesize approximately three times more ATP than they require for biomass synthesis, similar to the situation in Figure 5. The efficiency of ATP utilization in living cells is often approximately three-fold lower than would be predicted from standard biosynthetic costs. This is because of the existence of processes such as maintenance energy, futile cycling, ATP spilling, and uncoupling that consume ATP (or diminish synthesis) with no yield in terms of growth or cell mass (Russell and Cook, 1995; Russell, 2007; Hoehler and Jörgensen, 2013). The theoretical maximum yield in terms of net cell mass increase per ATP is always lower than the observed value in studies of modern cells (Stouthamer, 1978); it is also lower in Figure 5.

Primordial carbon metabolism involves successive incorporation of CO_2_ into acetyl CoA, pyruvate, oxaloacetate, and 2-oxoglutarate via the acetyl CoA pathway and incomplete reverse citric acid cycle. This conserved core provides the carbon backbones for the synthesis of amino acids, whereby amino acids (glycine, aspartate, glutamine) plus CO_2_ and C_1_ intermediates of the acetyl CoA pathway provide in turn the carbon backbones and nitrogen for the synthesis of purines and pyrimidines (Lipmann, 1965; Martin and Russell, 2007). Note that amino acid, sugar (for example, ribose), and nucleobase synthesis in microbial metabolism does not start from the successive incorporation of formaldehyde units (Ricardo et al., 2004), cyanide units (Canavelli et al., 2019), oxidized methane units (Nitschke and Russell, 2013), or acetate units, as one reader suggested. Rather, it starts with the successive incorporation of CO_2_ units (Figure 5), and is energetically financed in acetogens and methanogens by exergonic reactions of CO_2_ with H_2_.

If we step back for a moment, we recognize that the CO_2_-based design of central intermediary metabolism is a very, very strong argument in favor of autotrophic origins (carbon from CO_2_). Theories based in polymerization of formaldehyde, cyanide, activated methane, or acetate do not intersect at all with central metabolism of real cells, whereas theories based in the sequential condensation of CO_2_ do—seamlessly (Figure 5) and without corollary assumptions.

Figure 5 also underscores that synthesis of protein (the main substance of life) is a side reaction of a main exergonic reaction. It furthermore underscores the point that there is a kind of natural order in metabolism, as Morowitz (1968) suggested. Note that the line widths of gray arrows indicating carbon flux in Figure 5, also the large vertical one at left, are drawn roughly to scale relative to one another. The main reaction in the cell is bioenergetic. Cell mass is a byproduct.

If we keep thermodynamic constraints on metabolism in mind, it is evident that the vectors of evolutionary progression across the reactions in Figure 5 cannot start with ribosomes at the top right, because the energy-releasing reactions required for their synthesis start at the lower left, from H_2_ and CO_2_. For thermodynamic reasons, the vector of evolutionary progression in Figure 5 has to start at the bottom left. In autotrophic origins, the evolution of carbon pathways progresses from left to right and bottom to top, from simpler to complex. At the very beginning of evolution, small pathways had to start without enzymes and had to be exergonic (Preiner et al., 2020), autocatalytic reaction sets probably played a role as intermediates (Kauffman, 1986; Hordijk and Steel, 2004; Xavier et al., 2020), and, once genes arose, more specific biochemical pathways could evolve, probably in a patterned fashion, with naturally occurring chemical reactions paving the way of the evolution of the first metabolic pathways. But for all of that to occur, there had to be a continuous, uninterrupted energy-releasing reaction driving it all. H_2_-dependent CO_2_ reduction as it occurs at hydrothermal vents is the proposition.

The complex reactions moving left to right and bottom to top in Figure 5 are in many cases not sufficiently exergonic to go forward by themselves and hence require come kind of chemical connection, or coupling, to the main exergonic acetate-generating reaction. In this article, I have argued that energetic coupling first involved SLP (abiotic) to generate acyl phosphates in the course of continuous acetate synthesis from H_2_ and CO_2_ and later involved the harnessing of naturally preexisting proton gradients by the ATP synthase subsequent to the origin of genes. I made a similar case previously (Martin and Russell, 2007; Lane and Martin, 2012), but the case is now better backed by evidence. At origins, there had to be energy harnessing from the very start, because most of the reactions in metabolism are not strongly exergonic, and some are endergonic, requiring coupling to ATP (or similar) hydrolysis to move forward. Thus, evolution at the level of genes and pathways depends, from the very start of biochemical evolution, upon exergonic redox reactions of carbon (Martin and Thauer, 2017) during H_2_-dependent CO_2_ reduction to acetyl CoA and pyruvate. In Figure 5, as in the experiments of Preiner et al. (2020), acetate is synthesized via the intermediates of the acetyl CoA pathway.

## Conclusion

Autotrophic theories have a long tradition. Hydrogen-dependent acetogens figure centrally in modern autotrophic theory because the backbone of their metabolism, the acetyl CoA pathway, provides both carbon and energy from the H_2_-dependent synthesis of acetate from CO_2_. Of the six CO_2_ fixing pathways known, only the acetyl CoA pathway generates ATP; the other five require ATP input. Because primordial biochemical reactions had to be exergonic, this is a strong argument for antiquity of the acetyl CoA pathway. The unique involvement of CO as an intermediate in the pathway has the consequence that it generates carboxyls (acetate) from carbonyls (acetyl), whereas the other five pathways incorporate CO_2_ as carboxyls that have to be reduced to carbonyls at the cost of energy input. As with almost all forms of SLP, SLP in the acetogen pathway entails the nucleophilic attack of a carbonyl carbon in a thioester by an otherwise inert inorganic phosphate ion to generate an acyl phosphate that can phosphorylate ADP (Weiße et al., 2016). The energy in SLP thus stems from activated carbon atoms reacting with phosphate, not from compounds such as pyrophosphate, polyphosphates, phosphites, or phosphides reacting with unreactive carbon species. Because the reaction of H_2_ and CO_2_ continuously generates reactive carbonyl intermediates *en route* to free organic acids, the source of energy behind phosphate-based energy conservation at origins was most likely H_2_-dependent CO_2_ reduction, not reactive phosphorous minerals, counter to many traditional concepts about prebiotic chemistry. Primordial carbon metabolism consists of the sequential addition of CO_2_ molecules to form acetyl moieties, pyruvate, oxaloacetate, 2-oxoglutarate, and sugars, from which all 20 amino acids are ultimately derived. This strongly suggests that metabolism arose from CO_2_ in accordance with theories for autotrophic origins, as opposed to origins from other carbon species such as formaldehyde condensations, cyanide condensations, or methane oxidation. The central reactions of the acetyl CoA pathway to form formate, acetate, and pyruvate from H_2_ and CO_2_ take place overnight at 100°C without enzymes under hydrothermal vent conditions using only hydrothermal vent minerals as catalysts. This suggests that biochemical pathways evolved from CO_2_ outward via a process of patterned pathway evolution, in which naturally occurring anabolic chemical reactions paved the way for reactions that later came to be catalyzed by enzymes. Patterned evolution of pathways is the autotrophic counterpart and converse of retrograde pathway evolution in heterotrophic theories. Carbon and energy metabolism as they are manifest in the metabolism of acetogens (and methanogens) that lack cytochromes growing on H_2_ and CO_2_ likely reflects an ancestral state of microbial physiology from which the evolution of more complex pathways involving fermentations and cytochrome-dependent electron transport chains emerged.

## Author Contributions

WM wrote the manuscript and prepared the figures.

## Conflict of Interest

The author declares that the research was conducted in the absence of any commercial or financial relationships that could be construed as a potential conflict of interest.

## References

[B1] Akashi H. Gojobori T. (2002). Metabolic efficiency and amino acid composition in the proteomes of *Escherichia coli* and *Bacillus subtilis*. *Proc. Natl. Acad. Sci. U.S.A.* 99 3695–3700. 11904428 10.1073/pnas.062526999 PMC122586

[B2] Allen J. F. (2005). A redox switch hypothesis for the origin of two light reactions in photosynthesis. *FEBS Lett.* 579 963–968. 10.1016/J.Febslet.2005.01.015 15710376

[B3] Andreesen J. R. (2004). Glycine reductase mechanism. *Curr. Opin. Chem. Biol.* 8 454–461. 15450486 10.1016/j.cbpa.2004.08.002

[B4] Arndt N. T. Nisbet E. G. (2012). Processes on the young Earth and the habitats of early life. *Annu. Rev. Earth. Planet. Sci.* 40 521–549.

[B5] Bach W. Paulick H. Garrido C. J. Ildefonse B. Meurer W. P. Humphris S. (2006). Unraveling the sequence of serpentinization reactions: petrography, mineral chemistry, and petrophysics of serpentinites from MAR 15°N (ODP Leg 209, Site 1274). *Geophys. Res. Lett.* 33:L13306 10.1029/2006GL025681

[B6] Bada J. L. Lazcano A. (2002). Some like it hot, but not the first biomolecules. *Science* 296 1982–1983. 10.1126/science.1069487 12065824

[B7] Baross J. A. (2018). The rocky road to biomolecules. *Nature* 564 42–43. 10.1038/d41586-018-07262-8 30510224

[B8] Baross J. A. Hoffmann S. E. (1985). Submarine hydrothermal vents and associated gradient environments as sites for the origin and evolution of life. *Orig. Life Evol. Biosph.* 15 327–345. 10.1007/BF01808177

[B9] Basen M. Müller V. (2017). Hot” acetogenesis. *Extremophiles* 21 15–26. 10.1007/s00792-016-0873-3 27623994

[B10] Becker S. Schneider C. Crisp A. Carell T. (2018). Non-canonical nucleosides and chemistry of the emergence of life. *Nat. Comm.* 9:5174. 10.1038/s41467-018-07222-w 30538241 PMC6289997

[B11] Benner S. A. Kim H.-J. (2015). “The case for a martian origin for earth life,” in *Proceedings of the SPIE 9606, Instruments, Methods, and Missions for Astrobiology XVII, 96060C*, eds Hoover R. B. Levin G. V. Rozanov A. Y. Wickramasinghe N. C. (Bellingham, WA: SPIE), 10.1117/12.2192890

[B12] Berg I. A. (2011). Ecological aspects of the distribution of different autotrophic CO_2_ fixation pathways. *Appl. Environ. Microbiol.* 77 1925–1936. 21216907 10.1128/AEM.02473-10 PMC3067309

[B13] Berg I. A. Kockelkorn D. Ramos-Vera W. H. Say R. F. Zarzycki J. Hügler M. (2010). Autotrophic carbon fixation in archaea. *Nat. Rev. Microbiol.* 8 447–460. 10.1038/nrmicro2365 20453874

[B14] Berg J. M. Tymoczko J. L. Gatto G. J. Stryer L. (2015). *Biochemistry*, 8th Edn New York, NY: W. W. Freemann.

[B15] Boyd E. S. Amenabar M. J. Poudel S. Templeton A. S. (2019). Bioenergetic constraints on the origin of autotrophic metabolism. *Philos. Trans. R. Soc. A* 378:20190151. 10.1098/rsta.2019.0151 31902344 PMC7015307

[B16] Buckel W. Eggerer H. (1965). On the optical determination of citrate synthase and acetyl-coenzyme A. *Biochem. Z.* 343 29–43. 4289790

[B17] Buckel W. Thauer R. K. (2013). Energy conservation via electron bifurcating ferredoxin reduction and proton/Na^+^ translocating ferredoxin oxidation. *Biochim. Biophys. Acta Bioenergy* 1827 94–113. 10.1016/j.bbabio.2012.07.002 22800682

[B18] Buckel W. Thauer R. K. (2018). Flavin-based electron bifurcation, ferredoxin, flavodoxin, and anaerobic respiration with protons (Ech) or NAD^+^ (Rnf) as electron acceptors: a historical review. *Front. Microbiol.* 9:401. 10.3389/fmicb.2018.00401 29593673 PMC5861303

[B19] Canavelli P. Islam S. Powner M. W. (2019). Peptide ligation by chemoselective aminonitrile coupling in water. *Nature* 571 546–549. 31292542 10.1038/s41586-019-1371-4

[B20] Carter C. W. Wills P. R. (2019). Class I and II aminoacyl-tRNA synthetase tRNA groove discrimination created the first synthetase–tRNA cognate pairs and was therefore essential to the origin of genetic coding. *IUBMB Life* 71 1088–1098. 10.1002/iub.2094 31190358 PMC6642019

[B21] Corliss J. B. Baross J. A. Hoffmann S. E. (1981). An hypothesis concerning the relationship between submarine hot springs and the origin of life on Earth. *Oceanol. Acta* 4(Suppl.), 59–69.

[B22] Daniel S. L. Hsu T. Dean S. I. Drake H. L. (1990). Characterization of the H${}_{2}^{-}$ and CO-dependent chemolithotrophic potentials of the acetogens *Clostridium thermoaceticum* and *Acetogenium kivui*. *J. Bacteriol.* 172 4464–4471. 2376565 10.1128/jb.172.8.4464-4471.1990 PMC213276

[B23] Deamer D. Weber A. L. (2010). Bioenergetics and life’s origins. *Cold Spring Harb. Perspect. Biol.* 2:a004929. 10.1101/cshperspect.a004929 20182625 PMC2828274

[B24] Decker K. Jungermann K. Thauer R. K. (1970). Energy production in anaerobic organisms. *Angew. Chem. Int. Ed.* 9 138–158. 10.1002/anie.197001381 4984685

[B25] de Duve C. (1991). *Blueprint for a Cell: The Nature and Origin of Life.* Burlington, NC: Neil Patterson Publishers.

[B26] Di Giulio M. (2011). The last universal common ancestor (LUCA) and the ancestors of archaea and bacteria were progenotes. *J. Mol. Evol.* 72 119–126. 10.1007/s00239-010-9407-2 21079939

[B27] Dobbek H. (2018). “Metallocofactors that activate small molecules,” in *Structure and Bonding*, Vol. 179 ed. Ribbe M. W. (Cham: Springer International Publishing), 153 10.1007/430_2018_27

[B28] Dobbek H. Svetlitchnyi V. Gremer L. Huber R. Meyer O. (2001). Crystal structure of a carbon monoxide dehydrogenase reveals a [Ni-4Fe-5S] cluster. *Science* 293 1281–1285. 10.1126/science.1061500 11509720

[B29] Doukov T. I. Blasiak L. C. Seravalli J. Ragsdale S. W. Drennan C. L. (2008). Xenon in and at the end of the tunnel of bifunctional carbon monoxide dehydrogenase/acetyl-CoA synthase. *Biochemistry* 47 3474–3483. 10.1021/bi702386t 18293927 PMC3040099

[B30] Drake H. L. Gößner A. S. Daniel S. L. (2008). Old acetogens, new Light. *Ann. N. Y. Acad. Sci.* 1125 100–128. 10.1196/annals.1419.016 18378590

[B31] Drennan C. L. Doukov T. I. Ragsdale S. W. (2004). The metalloclusters of carbon monoxide dehydrogenase/acetyl-CoA synthase: a story in pictures. *J. Biol. Inorg. Chem.* 9 511–515. 10.1007/s00775-004-0563-y 15221484

[B32] Eck R. V. Dayhoff M. O. (1966). Evolution of the structure of ferredoxin based on living relics of primitive amino acid sequences. *Science* 152 363–366. 17775169 10.1126/science.152.3720.363

[B33] Etiope G. Schoell M. (2014). Abiotic gas: atypical, but not rare. *Elements* 10 291–296. 10.2113/gselements.10.4.291 28159795

[B34] Ferry J. G. House C. H. (2006). The stepwise evolution of early life driven by energy conservation. *Mol. Biol. Evol.* 23 1286–1292. 16581941 10.1093/molbev/msk014

[B35] Fischer W. W. Hemp J. Johnson J. E. (2016). Evolution of oxygenic photosynthesis. *Ann. Rev. Earth Planet Sci.* 44 647–683.

[B36] Frey P. A. Arabshahi A. (1995). Standard free energy change for the hydrolysis of the alpha, beta-phosphoanhydride bridge in ATP. *Biochemistry* 34 11307–11310. 7547856 10.1021/bi00036a001

[B37] Fuchs G. (2011). Alternative pathways of carbon dioxide fixation: insights into the early evolution of life? *Annu. Rev. Microbiol.* 65 631–658. 10.1146/annurev-micro-090110-102801 21740227

[B38] Fuchs G. Stupperich E. (1985). “Evolution of autotrophic CO2 fixation,” in *Evolution of Prokaryotes*, eds Schleifer K. H. Stackebrandt E. (London: Academic Press), 235–225.

[B39] Garrison W. M. Morrison D. C. Hamilton J. G. Benson A. A. Calvin M. (1951). Reduction of carbon dioxide in aqueous solutions by ionizing radiation. *Science* 114 416–418. 10.1126/science.114.2964.416 14892746

[B40] Goldford J. E. Hartman H. Marsland R. Segrè D. (2019). Environmental boundary conditions for the origin of life converge to an organo-sulfur metabolism. *Nat. Ecol. Evol.* 3 1715–1724. 10.1038/s41559-019-1018-8 31712697 PMC6881557

[B41] Goldford J. E. Hartman H. Smith T. F. Segre D. (2017). Remnants of an ancient metabolism without phosphate. *Cell* 168 1126–1134. 28262353 10.1016/j.cell.2017.02.001

[B42] Graham D. E. White R. H. (2002). Elucidation of methanogenic coenzyme biosyntheses: from spectroscopy to genomics. *Nat. Prod. Rep.* 19 133–147. 10.1039/b103714p 12013276

[B43] Grüber G. Manimekalai M. S. S. Mayer F. Müller V. (2014). ATP synthases from archaea: the beauty of a molecular motor. *Biochim. Biophys. Acta Bioenergy* 1837 940–952. 10.1016/j.bbabio.2014.03.004 24650628

[B44] Haeckel E. (1902). *Natürliche Schöpfungs-Geschichte. Gemeinverständliche Wissenschaftliche Vorträge über die Entwickelungslehre Zehnte verbesserte Auflage. Zweiter Theil: Allgemeine Stammesgeschichte* (Berlin: Georg Reimer Verlag).

[B45] Haldane J. B. S. (1929). The origin of life. *Ration. Annu.* 148 3–10.

[B46] Hall D. O. Cammack R. Rao K. K. (1971). Role of ferredoxins in the origin of life and biological evolution. *Nature* 233 136–138. 12058758 10.1038/233136a0

[B47] Hansen L. D. Criddle R. S. Battley E. H. (2009). Biological calorimetry and the thermodynamics of the origination and evolution of life. *Pure Appl. Chem.* 81 1843–1855. 10.1351/PAC-CON-08-09-09

[B48] Hattori S. Galushko A. S. Kamagata Y. Schink B. (2005). Operation of the CO dehydrogenase/acetyl coenzyme A pathway in both acetate oxidation and acetate formation by the syntrophically acetate-oxidizing bacterium *Thermacetogenium phaeum*. *J. Bacteriol.* 187 3471–3476. 15866934 10.1128/JB.187.10.3471-3476.2005 PMC1111993

[B49] Herrmann G. Jayamani E. Mai G. Buckel W. (2008). Energy conservation via electron-transferring flavoprotein in anaerobic bacteria. *J. Bacteriol.* 190 784–791. 10.1128/JB.01422-07 18039764 PMC2223574

[B50] Hess V. Poehlein A. Weghoff M. C. Daniel R. Müller V. (2014). A genome-guided analysis of energy conservation in the thermophilic, cytochrome-free acetogenic bacterium *Thermoanaerobacter kivui*. *BMC Genomics* 15:1139. 10.1186/1471-2164-15-1139 25523312 PMC4320612

[B51] Hoehler T. M. Jörgensen B. B. (2013). Microbial life under extreme energy limitation. *Nat. Rev. Microbiol.* 2013 83–94. 10.1038/nrmicro2939 23321532

[B52] Hordijk W. Steel M. (2004). Detecting autocatalyctic, self-sustaining sets in chemical reaction systems. *J. Theor. Biol.* 227 451–461. 15038982 10.1016/j.jtbi.2003.11.020

[B53] Horowitz N. H. (1945). On the evolution of biochemical syntheses. *Proc. Natl. Acad. Sci U.S.A.* 31 153–157. 16578152 10.1073/pnas.31.6.153 PMC1078786

[B54] Huang G. Wagner T. Ermler U. Shima S. (2020). Methanogenesis involves direct hydride transfer from H_2_ to an organic substrate. *Nat. Rev. Chem.* 4 213–221. 10.1038/s41570-020-0167-2 37128042

[B55] Hügler M. Sievert S. M. (2011). Beyond the calvin cycle: autotrophic carbon fixation in the ocean. *Annu. Rev. Mar. Sci.* 3 261–289. 10.1146/annurev-marine-120709-142712 21329206

[B56] Katchalsky A. Paecht M. (1954). Phosphate anhydrides of amino Acids. *J. Biol Chem.* 76 6042–6044.

[B57] Kauffman S. A. (1986). Autocatalytic sets of proteins. *J. Theor. Biol.* 19 1–24. 10.1016/s0022-5193(86)80047-9 3713221

[B58] Keller M. A. Piedrafita G. Ralser M. (2015). The widespread role of non-enzymatic reactions in cellular metabolism. *Curr. Opin. Biotechnol.* 34 153–161. 10.1016/j.copbio.2014.12.020 25617827 PMC4728180

[B59] Keller M. A. Turchyn A. V. Ralser M. (2014). Non-enzymatic glycolysis and pentose phosphate pathway-like reactions in a plausible Archean ocean. *Mol. Syst. Biol.* 10:725. 10.1002/msb.20145228 24771084 PMC4023395

[B60] Kitani A. Tsunetsugu S. Sasaki K. (1991). FeIII-ion-catalyzed nonenzymatic transformation of ADP into ATP. *J. Chem. Soc. Perkin Trans.* 2 329–331.

[B61] Kitani A. Tsunetsugu S. Suzuki A. Ito S. Sasaki K. (1995). Fe(III)-ion-catalyzed nonenzymatic transformation of adenosine-diphosphate into adenosine-triphosphate. 2. Evidence of catalytic nature of Fe ions. *Bioelectrochem. Bioenerget.* 36 47–51.

[B62] Krishnaro J. S. R. (1964). Native nickel-iron alloy, its mode of occurrence, distribution and origin. *Econ. Geol.* 59 443–448.

[B63] Lane N. Allen J. F. Martin W. F. (2010). How did LUCA make a living? Chemiosmosis in the origin of life. *BioEssays* 32 271–280. 10.1002/bies.200900131 20108228

[B64] Lane N. Martin W. F. (2012). The origin of membrane bioenergetics. *Cell* 151 1406–1416. 10.1016/j.cell.2012.11.050 23260134

[B65] Lang S. Q. Fruh-Green G. L. Bernasconi S. M. Brazelton W. J. Schrenk M. O. McGonigle J. M. (2018). Deeply-sourced formate fuels sulfate reducers but not methanogens at Lost City hydrothermal field. *Sci. Rep.* 8:755. 10.1038/s41598-017-19002-5 PMC5768773 29335466

[B66] Lipmann F. (1941). Metabolic generation and utilization of phosphate bond energy. *Adv. Enzymol.* 1 99–162. 10.1002/9780470122464.ch4

[B67] Lipmann F. (1965). “Projecting backward from the present stage of evolution of biosynthesis,” in *The Origin of Prebiological Systems and of their Molecular Matrices*, ed. Fox S. W. (New York, NY: Academic Press), 259–280.

[B68] Liu Y. C. Beer L. L. Whitman W. B. (2012). Methanogens: a window into ancient sulfur metabolism. *Trends. Microbiol.* 20 251–258. 22406173 10.1016/j.tim.2012.02.002

[B69] Ljungdahl L. G. (2009). A life with acetogens, thermophiles, and cellulolytic anaerobes. *Annu. Rev. Microbiol.* 63 1–25. 10.1146/annurev.micro.091208.073617 19575555

[B70] Maden B. E. (1995). No soup for starters? Autotrophy and the origins of metabolism. *Trends Biochem. Sci.* 20 337–341. 10.1016/s0968-0004(00)89069-6 7482696

[B71] Maden B. E. H. (2000). Tetrahydrofolate and tetrahydromethanopterin compared: functionally distinct carriers in C1 metabolism. *Biochem. J.* 350 609–629. 10.1042/bj3520935u 10970772 PMC1221290

[B72] Madigan M. T. Martinko J. M. Bender K. S. Buckley D. H. Stahl D. A. (2019). *Brock Biology of Microorganisms*, 15th Edn Harlow: Pearson Education.

[B73] Magnabosco C. Lin L.-H. Dong H. Bomberg M. Ghiorse W. Stan-Lotter H. (2018). The biomass and biodiversity of the continental subsurface. *Nat. Geosci.* 11 707–717. 10.1038/s41561-018-0221-6

[B74] Mall A. Sobotta J. Huber C. Tschirner C. Kowarschik S. Bačnik K. (2018). Reversibility of citrate synthase allows autotrophic growth of a thermophilic bacterium. *Science* 359 563–567. 10.1126/science.aao2410 29420287

[B75] Martin W. (2008). On the ancestral state of microbial physiology. In Amann R, Goebel W, Schink B, Widdel F (Ed) life strategies of microorganisms in the environment and in host organisms. *Nova Acta Leopoldina* 96 53–60.

[B76] Martin W. Russell M. J. (2003). On the origins of cells: a hypothesis for the evolutionary transitions from abiotic geochemistry to chemoautotrophic prokaryotes, and from prokaryotes to nucleated cells. *Philos. Trans. R. Soc. Lond. B Biol. Sci.* 358 59–83. 12594918 10.1098/rstb.2002.1183 PMC1693102

[B77] Martin W. F. (2012). Hydrogen, metals, bifurcating electrons, and proton gradients: the early evolution of biological energy conservation. *FEBS Lett.* 586 485–493. 10.1016/j.febslet.2011.09.031 21978488

[B78] Martin W. F. (2019). Carbon metal bonds, rare and primordial in metabolism. *Trends Biochem. Sci.* 44 807–818. 31104860 10.1016/j.tibs.2019.04.010

[B79] Martin W. F. Bryant D. A. Beatty J. T. (2018). A physiological perspective on the origin and evolution of photosynthesis. *FEMS Microbiol. Rev.* 42 201–231. 10.1093/femsre/fux056 PMC5972617 29177446

[B80] Martin W. F. Cerff R. (2017). Physiology, phylogeny, early evolution, and GAPDH. *Protoplasma* 254 1823–1834. 10.1007/s00709-017-1095-y 28265765 PMC5610209

[B81] Martin W. F. Russell M. J. (2007). On the origin of biochemistry at an alkaline hydrothermal vent. *Philos. Trans. R. Soc. B* 362 1887–1925. 10.1098/rstb.2006.1881 17255002 PMC2442388

[B82] Martin W. F. Sousa F. L. (2015). Early microbial evolution: the age of anaerobes. *Cold Spring Harb. Perspect. Biol.* 8:a018127. 10.1101/cshperspect.a018127 26684184 PMC4743081

[B83] Martin W. F. Sousa F. L. (2016). Early microbial evolution: the age of anaerobes. *Cold Spring Harb. Perspect. Biol.* 8:a018127. 10.1101/cshperspect.a018127 26684184 PMC4743081

[B84] Martin W. F. Thauer R. K. (2017). Energy in ancient metabolism. *Cell* 168 953–955. 10.1016/j.cell.2017.02.032 28283068

[B85] Mayumi D. Mochimaru H. Tamaki H. Yamamoto K. Yoshioka H. Suzuki Y. (2016). Methane production from coal by a single methanogen. *Science* 354 222–225. 10.1126/science.aaf8821 27738170

[B86] Ménez B. Pisapia C. Andreani M. Jamme F. Vanbellingen Q. P. Brunelle A. (2018). Abiotic synthesis of amino acids in the recesses of the oceanic lithosphere. *Nature* 564 59–63. 30405236 10.1038/s41586-018-0684-z

[B87] Mereschkowsky C. (1910). Theorie der zwei Plasmaarten als Grundlage der Symbiogenesis, einer neuen Lehre von der Entstehung der Organismen. *Biol. Centralbl.* 30 278–288, 289–303, 353–367.

[B88] Miller S. L. Urey H. (1959). Organic compound synthesis on the primitive Earth. *Science* 130 245–251. 10.1126/science.130.3370.245 13668555

[B89] Mitchell P. (1961). Coupling of phosphorylation to electron and hydrogen transfer by a chemi-osmotic type of mechanism. *Nature* 191 144–148. 10.1038/191144a0 13771349

[B90] Morowitz H. J. (1968). *Energy Flow in Biology.* New York, NY: Academic Press.

[B91] Morowitz H. J. (1992). *Beginnings of Cellular Life: Metabolism Recapitulates Biogenesis.* New Haven, CT: Yale University Press.

[B92] Muchowska K. B. Varma S. J. Chevallot-Berou E. Lethuillier-Karl L. Li G. Moran J. (2017). Metals promote sequences of the reverse Krebs cycle. *Nature Ecol. Evol.* 1 1716–1721. 28970480 10.1038/s41559-017-0311-7 PMC5659384

[B93] Muchowska K. B. Varma S. J. Moran J. (2019). Synthesis and breakdown of universal metabolic precursors promoted by iron. *Nature* 569 104–107. 31043728 10.1038/s41586-019-1151-1 PMC6517266

[B94] Müller V. Chowdhury N. P. Basen M. (2018). Electron bifurcation: a long-hidden energy-coupling mechanism. *Annu. Rev. Microbiol.* 72 331–353. 10.1146/annurev-micro-090816-093440 29924687

[B95] Nelson-Sathi S. Dagan T. Landan G. Janssen A. Steel M. McInerney J. O. (2012). Acquisition of 1,000 eubacterial genes physiologically transformed a methanogen at the origin of Haloarchaea. *Proc. Natl. Acad. Sci. U.S.A.* 109 20537–20542. 23184964 10.1073/pnas.1209119109 PMC3528564

[B96] Nelson-Sathi S. Sousa F. L. Roettger M. Lozada-Chávez N. Thiergart T. Janssen A. (2015). Origins of major archaeal clades correspond to gene acquisitions from bacteria. *Nature* 517 77–80. 25317564 10.1038/nature13805 PMC4285555

[B97] Nisbet E. G. Cann J. R. Van Dover C. L. (1995). Origins of photosynthesis. *Nature* 373 479–480.

[B98] Nisbet E. G. Sleep N. H. (2001). The habitat and nature of early life. *Nature* 409 1083–1091. 11234022 10.1038/35059210

[B99] Nitschke W. Russell M. J. (2013). Beating the acetyl coenzyme A-pathway to the origin of life. *Philos Trans. R. Soc. Lond. B* 368:20120258. 10.1098/rstb.2012.0258 PMC3685460 23754811

[B100] Nunoura T. Chikaraishi Y. Izaki R. Suwa T. Sato T. Harada T. (2018). A primordial and reversible TCA cycle in a facultatively chemolithoautotrophic thermophile. *Science* 359 559–563. 10.1126/science.aao3407 29420286

[B101] Orgel L. E. (2008). The implausibility of metabolic cycles on the prebiotic earth. *PLoS Biol.* 6:e18. 10.1371/journal.pbio.0060018 18215113 PMC2211548

[B102] Pasek M. A. Gull M. Herschy B. (2017). Phosphorylation on the early earth. *Chem. Geol.* 475 149–170. 10.1016/j.chemgeo.2017.11.008

[B103] Peters J. W. Beratan D. N. Bothner B. Dyer R. B. Harwood C. S. Heiden Z. M. (2018). A new era for electron bifurcation. *Curr. Opin. Chem. Biol.* 47 32–38. 10.1016/j.cbpa.2018.07.026 30077080 PMC9113080

[B104] Preiner M. Igarashi K. Muchowska K. B. Yu M. Varma S. J. Kleinermanns K. (2020). A hydrogen dependent geochemical analogue of primordial carbon and energy metabolism. *Nat. Ecol. Evol.* 4 534–542. 32123322 10.1038/s41559-020-1125-6

[B105] Preiner M. Xavier J. Sousa F. Zimorski V. Neubeck A. Lang S. Q. (2018). Serpentinization: connecting geochemistry, ancient metabolism and industrial hydrogenation. *Life* 8:41. 10.3390/life8040041 PMC6316048 30249016

[B106] Proskurowski G. Lilley M. D. Seewald J. S. Früh-Green G. L. Olson E. J. Lupton J. E. (2008). Abiogenic hydrocarbon production at lost city hydrothermal field. *Science* 319 604–607. 10.1126/science.1151194 18239121

[B107] Rabus R. Venceslau S. S. Wohlbrand L. Voordouw G. Wall J. D. Pereira I. A. C. (2015). “A post-genomic view of the ecophysiology, catabolism and biotechnological relevance of sulphate-reducing prokaryotes,” in *Advances in Microbial Physiology*, Vol. 66 ed. Poole R. K. (Oxford: Academic Press), 55–321. 10.1016/bs.ampbs.2015.05.002 26210106

[B108] Ragsdale S. W. (2004). Life with carbon monoxide. *Crit. Rev. Biochem. Mol. Biol.* 39 165–195. 10.1080/10409230490496577 15596550

[B109] Ragsdale S. W. (2008). Enzymology of the Wood-Ljungdahl pathway of acetogenesis. *Ann. N. Y. Acad. Sci.* 1125 129–136. 10.1196/annals.1419.015 18378591 PMC3040112

[B110] Ragsdale S. W. (2009). Nickel-based enzyme systems. *J. Biol. Chem.* 284 18571–18575. 19363030 10.1074/jbc.R900020200 PMC2707248

[B111] Ragsdale S. W. Pierce E. (2008). Acetogenesis and the Wood-Ljungdahl pathway of CO_2_ fixation. *Biochim. Biophys. Acta* 1784 1873–1898. 10.1016/j.bbapap.2008.08.012 18801467 PMC2646786

[B112] Ricardo A. Carrigan M. A. Olcott A. N. Benner S. A. (2004). Borate minerals stabilize ribose. *Science* 303 196. 10.1126/science.1092464 14716004

[B113] Russell J. B. (2007). The energy spilling reactions of bacteria and other organisms. *J. Mol. Microbiol. Biotechnol.* 13 1–11. 10.1159/000103591 17693707

[B114] Russell J. B. Cook G. M. (1995). Energetics of bacterial growth: balance of anabolic and catabolic reactions. *Microbiol. Rev.* 59 48–62. 7708012 10.1128/mr.59.1.48-62.1995 PMC239354

[B115] Russell M. J. (2006). First life. *Am. Sci.* 94 32–39. 10.1511/2006.57.32

[B116] Russell M. J. Hall A. J. (1997). The emergence of life from iron monosulphide bubbles at a submarine hydrothermal redox and pH front. *J. Geol. Soc. Lond.* 154 377–402. 11541234 10.1144/gsjgs.154.3.0377

[B117] Russell M. J. Martin W. (2004). The rocky roots of the acetyl-CoA pathway. *Trends Biochem. Sci.* 29 358–363. 15236743 10.1016/j.tibs.2004.05.007

[B118] Russell M. J. Hall A. J. Martin W. (2010). Serpentinization as a source of energy at the origin of life. *Geobiology* 8 355–371. 10.1111/j.1472-4669.2010.00249.x 20572872

[B119] Say R. F. Fuchs G. (2010). Fructose 1,6-bisphosphate aldolase/phosphatase may be an ancestral gluconeogenic enzyme. *Nature* 464 1077–1081. 10.1038/nature08884 20348906

[B120] Schauder R. Preuß A. Jetten M. Fuchs G. (1988). Oxidative and reductive acetyl CoA/carbon monoxide dehydrogenase pathway in *Desulfobacterium autotrophicum*. 2. Demonstration of the enzymes of the pathway and comparison of CO dehydrogenase. *Arch. Microbiol.* 151 84–89.

[B121] Schink B. Friedrich M. (2000). Phosphite oxidation by sulphate reduction. *Nature* 406:37. 10.1038/35017644 10894531

[B122] Schoelmerich M. C. Müller V. (2019). Energy conservation by a hydrogenase-dependent chemiosmotic mechanism in an ancient metabolic pathway. *Proc. Natl. Acad. Sci. U.S.A.* 116 6329–6334. 10.1073/pnas.1818580116 30850546 PMC6442639

[B123] Schönheit P. Buckel W. Martin W. (2016). On the origin of heterotrophy. *Trends Microbiol.* 24 12–25. 10.1016/j.tim.2015.10.003 26578093

[B124] Schuchmann K. Müller V. (2014). Autotrophy at the thermodynamic limit of life: a model for energy conservation in acetogenic bacteria. *Nat. Rev. Microbiol.* 12 809–821. 25383604 10.1038/nrmicro3365

[B125] Schuchmann K. Müller V. (2013). Direct and reversible hydrogenation of CO_2_ to formate by a bacterial carbon dioxide reductase. *Science* 342 1382–1385. 10.1126/science.1244758 24337298

[B126] Schut G. J. Boyd E. S. Peters J. W. Adams M. W. W. (2013). The modular respiratory complexes involved in hydrogen and sulfur metabolism by heterotrophic hyperthermophilic archaea and their evolutionary implications. *FEMS Microbiol. Rev.* 37 182–203. 10.1111/j.1574-6976.2012.00346.x 22713092

[B127] Semenov S. N. Kraft L. J. Ainla A. Zhao M. Baghbanzadeh M. Campbell V. E. (2016). Autocatalytic, bistable, oscillatory networks of biologically relevant organic reactions. *Nature* 537 656–660. 27680939 10.1038/nature19776

[B128] Shapiro R. (2006). Small molecule interactions were central to the origin of life. *Q. Rev. Biol.* 81 105–126. 10.1086/506024 16776061

[B129] Sleep N. H. Bird D. K. Pope E. C. (2011). Serpentinite and the dawn of life. *Philos. Trans. R. Soc. B* 366 2857–2869. 10.1098/rstb.2011.0129 21930576 PMC3158911

[B130] Sojo V. Pomiankowski A. Lane N. (2014). A bioenergetic basis for membrane divergence in archaea and bacteria. *PLoS Biol.* 12:e1001926. 10.1371/journal.pbio.1001926 25116890 PMC4130499

[B131] Sousa F. Martin W. F. (2014). Biochemical fossils of the ancient transition from geoenergetics to bioenergetics in prokaryotic one carbon compound metabolism. *Biochim. Biophys. Acta* 1837 964–981. 10.1016/j.bbabio.2014.02.001 24513196

[B132] Sousa F. L. Hordijk W. Steel M. Martin W. F. (2015). Autocatalytic sets in *E. coli* metabolism. *J. Systems Chem.* 6:4. 10.1186/s13322-015-0009-7 PMC4429071 25995773

[B133] Sousa F. L. Preiner M. Martin W. F. (2018). Native metals, electron bifurcation and CO_2_ reduction in early biochemical evolution. *Curr. Opin. Microbiol.* 43 77–83. 10.1016/j.mib.2017.12.010 29316496

[B134] Sousa F. L. Thiergart T. Landan G. Nelson-Sathi S. Pereira I. A. C. Allen J. F. (2013). Early bioenergetic evolution. *Philos. Trans. R. Soc. B* 368:20130088. 10.1098/rstb.2013.0088 23754820 PMC3685469

[B135] Stouthamer A. H. (1973). A theoretical study on the amount of ATP required for synthesis of microbial cell material. *Antonie Leeuwenhoek* 39 545–565. 4148026 10.1007/BF02578899

[B136] Stouthamer A. H. (1978). “Energy-yielding pathways,” in *The Bacteria Vol VI: Bacterial Diversity*, ed. Gunsalus I. C. (New York, NY: Academic Press).

[B137] Suzuki S. Nealson K. H. Ishii S. (2018). Genomic and in-situ transcriptomic characterization of the candidate phylum NPL-UPL2 from highly alkaline highly reducing serpentinized groundwater. *Front. Microbiol.* 9:3141. 10.3389/fmicb.2018.03141 30619209 PMC6305446

[B138] Svetlitchnaia T. Svetlitchnyi V. Meyer O. Dobbek H. (2006). Structural insights into methyltransfer reactions of a corrinoid iron-sulfur protein involved in acetyl-CoA synthesis. *Proc. Natl. Acad. Sci. U.S.A.* 103 14331–14336. 16983091 10.1073/pnas.0601420103 PMC1599964

[B139] Tashiro T. Ishida A. Hori M. Igisu M. Koike M. Méjean P. (2017). Early trace of life from 3.95 Ga sedimentary rocks in Labrador, Canada. *Nature* 549 516–518. 10.1038/nature24019 28959955

[B140] Thauer R. K. (2011). Hydrogenases and the global H_2_ cycle. *Eur. J. Inorg. Chem.* 2011 919–921.

[B141] Thauer R. K. (2015). My lifelong passion for biochemistry and anaerobic microorganisms. *Annu. Rev. Microbiol.* 69 1–30. 10.1146/annurev-micro-091014-104344 26488272

[B142] Thauer R. K. Jungermann K. Decker K. (1977). Energy conservation in chemotrophic anaerobic bacteria. *Bacteriol. Rev.* 41 100–180. 860983 10.1128/br.41.1.100-180.1977 PMC413997

[B143] Thauer R. K. Kaster A.-K. Seedorf H. Buckel W. Hedderich R. (2008). Methanogenic archaea: ecologically relevant differences in energy conservation. *Nat. Rev. Microbiol.* 6 579–591. 10.1038/nrmicro1931 18587410

[B144] Ueno Y. Yamada K. Yoshida N. Maruyama S. Isozaki Y. (2006). Evidence from fluid inclusions for microbial methanogenesis in the early Archaean era. *Nature* 440 516–519. 10.1038/nature04584 16554816

[B145] Valas R. E. Bourne P. E. (2011). The origin of a derived superkingdom: how a gram-positive bacterium crossed the desert to become an archaeon. *Biol. Direct.* 6:16. 10.1186/1745-6150-6-16 21356104 PMC3056875

[B146] Varma S. J. Muchowska K. B. Chatelain P. Moran J. (2018). Native iron reduces CO_2_ to intermediates and endproducts of the acetyl-CoA pathway. *Nat. Ecol. Evol.* 2 1019–1024. 10.1038/s41559-018-0542-2 29686234 PMC5969571

[B147] Volbeda A. Fontecilla-Camps J. C. (2006). Catalytic nickel–iron–sulfur clusters: from minerals to enzymes. *Top. Organomet. Chem.* 17 57–82. 10.1007/3418_003

[B148] Wächtershäuser G. (1992). Groundworks for an evolutionary biochemistry: the iron-sulphur world. *Prog. Biophys. Mol. Biol.* 58 85–201. 1509092 10.1016/0079-6107(92)90022-x

[B149] Wagner A. Whitaker R. J. Krause D. J. Heilers J. H. van Wolferen M. van der Does C. (2017). Mechanisms of gene flow in archaea. *Nat. Rev. Microbiol.* 2017 492–501. 10.1038/nrmicro.2017.41 28502981

[B150] Wagner T. Ermler U. Shima S. (2016). The methanogenic CO_2_ reducing-and-fixing enzyme is bifunctional and contains 46 [4Fe-4S] clusters. *Science* 354 114–117. 10.1126/science.aaf9284 27846502

[B151] Wald G. (1962). “Life in the second and third periods; or why phosphorus and sulfur for high-energy bonds?,” in *Horizons in Biochemistry*, eds Kasha M. Pullman B. (New York, NY: Academic Press), 127–142.

[B152] Wald G. (1964). The origins of life. *Proc. Natl. Acad. Sci. U.S.A.* 52 595–611. 10.1073/pnas.52.2.595 16591211 PMC300313

[B153] Weiss M. Preiner M. Xavier J. C. Zimorski V. Martin W. F. (2018). The last universal common ancestor between ancient Earth chemistry and the onset of genetics. *PLoS Genet*. 14:e1007518. 10.1371/journal.pgen.1007518 30114187 PMC6095482

[B154] Weiss M. C. Sousa F. L. Mrnjavac N. Neukirchen S. Roettger M. Nelson-Sathi S. (2016). The physiology and habitat of the last universal common ancestor. *Nat. Microbiol.* 1:16116. 10.1038/nmicrobiol.2016.116 27562259

[B155] Weiße R. H. Faust A. Schmidt M. Schönheit P. Scheidig A. J. (2016). Structure of NDP-forming Acetyl-CoA synthetase ACD1 reveals a large rearrangement for phosphoryl transfer. *Proc. Natl. Acad. Sci. U.S.A.* 113 E519–E528. 10.1073/pnas.1518614113 26787904 PMC4747732

[B156] Whicher A. Camprubi E. Pinna S. Herschy B. Lane N. (2018). Acetyl phosphate as a primordial energy currency at the origin of life. *Orig. Life Evol. Biosph.* 48 159–179. 29502283 10.1007/s11084-018-9555-8 PMC6061221

[B157] White R. H. (2001). Biosynthesis of the methanogenic cofactors. *Vitam. Horm.* 61 299–337. 10.1016/s0083-6729(01)61010-0 11153270

[B158] Wolfenden R. (2011). Benchmark reaction rates, the stability of biological molecules in water, and the evolution of catalytic power in enzymes. *Annu. Rev. Biochem.* 80 645–667. 21495848 10.1146/annurev-biochem-060409-093051

[B159] Wongnate T. Sliwa D. Ginovska B. Smith D. Wolf M. W. Lehnert N. (2016). The radical mechanism of biological methane synthesis by methyl-coenzyme M reductase. *Science* 352 953–958. 27199421 10.1126/science.aaf0616

[B160] Wood H. G. (1991). Life with CO or CO_2_ and H_2_ as a source of carbon and energy. *FASEB J.* 5 165–163. 10.1096/fasebj.5.2.1900793 1900793

[B161] Xavier J. C. Hordijk W. Kauffman S. Steel M. Martin W. F. (2020). Autocatalytic chemical networks preceded proteins and RNA in evolution. *Proc. R. Soc. Lond. B* 287. 10.1098/rspb.2019.2377 PMC7126077 32156207

[B162] Xavier J. C. Preiner M. Martin W. F. (2018). Something special about CO-dependent CO_2_ fixation. *FEBS J.* 285 4181–4195. 10.1111/febs.14664 30240136 PMC6282760

[B163] Ycas M. (1974). On earlier states of the biochemical system. *J. Theor. Biol.* 44 145–160. 4207200 10.1016/s0022-5193(74)80035-4

[B164] Zinder S. H. (1994). “Syntrophic acetate oxidation and “reversible acetogenesis”,” in *Acetogenesis*, ed. Drake H. L. (New York, NY: Chapman and Hall), 386–415.

